# Construction of a High-Density Genetic Map Based on Large-Scale Marker Development in Mango Using Specific-Locus Amplified Fragment Sequencing (SLAF-seq)

**DOI:** 10.3389/fpls.2016.01310

**Published:** 2016-08-30

**Authors:** Chun Luo, Bo Shu, Quangsheng Yao, Hongxia Wu, Wentian Xu, Songbiao Wang

**Affiliations:** Key Laboratory of Tropical Fruit Biology, Ministry of Agriculture; South Subtropical Crops Research Institute, Chinese Academy of Tropical Agricultural SciencesZhanjiang, China

**Keywords:** *Mangifera indica* L., SLAF-seq, high-density genetic map, SNPs, linkage group

## Abstract

Genetic maps are particularly important and valuable tools for quantitative trait locus (QTL) mapping and marker assisted selection (MAS) of plant with desirable traits. In this study, 173 F_1_ plants from a cross between *Mangifera indica* L. “Jin-Hwang” and *M. indica* L. “Irwin” and their parent plants were subjected to high-throughput sequencing and specific-locus amplified fragment (SLAF) library construction. After preprocessing, 66.02 Gb of raw data containing 330.64 M reads were obtained. A total of 318,414 SLAFs were detected, of which 156,368 were polymorphic. Finally, 6594 SLAFs were organized into a linkage map consisting of 20 linkage groups (LGs). The total length of the map was 3148.28 cM and the average distance between adjacent markers was 0.48 cM. This map could be considered, to our knowledge, the first high-density genetic map of mango, and might form the basis for fine QTL mapping and MAS of mango.

## Introduction

Mango (*Mangifera indica* L., 2*n* = 40) is a major fruit crop of the tropics and subtropics, and is often called the “king of fruits” (Mukherjee, 1950, 1957; Purseglove, 1972). Mango has been referred to as an allopolyploid (Mukherjee, 1950), but the recent microsatellite marker studies of Duval et al. (2005), Viruel et al. (2005), and Schnell et al. (2005) indicate that *M. indica* is diploid. Although mango has a high level of heterozygosity, it has a relatively small genome size (approximately 439 Mb; Arumuganathan and Earle, 1991). In recent years, the area under mango plantation has expanded unceasingly worldwide, and mango yields have increased year by year. Currently, more than 100 countries and regions worldwide produce mango, and China is one of the major mango-producing countries. The mango industry has become the mainstay of the local agricultural industry in most of the mango production provinces in China.

Mango breeding has gained increasing attention in recent years, and conventional breeding methods (cross breeding, mutation breeding, and seedling selection) are mainly used for creating new varieties of mango. However, these methods are time-consuming and difficult to improve upon because of some inherent characteristics of mango (Iyer and Schnell, 2009). Many important economic traits of mango are controlled by a number of loci. Quantitative trait locus (QTL) analysis and marker assisted selection (MAS) can shorten selection times and accelerate the breeding process of new varieties of mango. Genetic maps, especially high-density genetic maps, are particularly valuable tools for QTL mapping and MAS. Four genetic maps of mango have been generated to date. Chunwongse et al. (2000) first constructed a genetic map of mango using 197 restriction fragment length polymorphism (RFLP) and 650 amplified fragment length polymorphism (AFLP) markers based on 31 F_1_ plants from a cross between Alphonso and Palmer cultivars of mango (*M. indica*). Kashkush et al. (2001) developed the second genetic map of mango using 34 AFLP markers based on 29 F_1_ individuals and covering a length of 161.5 cM with an average marker spacing of 4.75 cM. Fang et al. (2003) developed the third genetic map of mango using 81 AFLP markers based on 60 F_1_ individuals and covering a length of 354.1 cM with an average marker spacing of 4.37 cM. Chunwongse et al. (2015) constructed a partial genetic linkage map spanning a distance of 529.9 cM and consisting of 9 microsatellite and 67 RFLP markers using the same plant materials as in their previous report (Chunwongse et al., 2000). However, these maps were developed on the basis of either a small mapping population (60 or fewer) or a limited number of markers, resulting in a relatively low-density genetic map for future QTL analysis.

DNA markers such as AFLP, RFLP, random amplification of polymorphic DNA (RAPD), inter simple sequence repeat (ISSR), sequence-related amplified polymorphism (SRAP), and simple sequence repeat (SSR) have been developed in mango. However, the current number of markers is too small to build a high-density genetic map. Specific-locus amplified fragment sequencing (SLAF-seq) technology is an efficient method of *de novo* single nucleotide polymorphism (SNP) discovery and large-scale genotyping, which is based on reduced-representation library (RRL) and high-throughput sequencing (Sun et al., 2013). The efficiency of SLAF-seq was tested using rice and soybean data, and has been used to construct the highest-density genetic map of common carp, without a reference genome sequence. To date, SLAF-seq has been used successfully to construct high-density genetic maps and study the genomes of many crops (Chen et al., 2013; Huang et al., 2013; Zhang et al., 2013, 2015; Li et al., 2014; Qi et al., 2014; Wei et al., 2014; Guo et al., 2015; Jiang et al., 2015; Xu et al., 2015; Zhu et al., 2015).

We conducted a hybridization breeding study of mango 10 years ago and established many segregating populations. Following several years' field investigation, we selected an intraspecific cross of *M. indica* for constructing its genetic linkage map. We employed the recently developed SLAF-seq approach to identify a large number of SNP markers for mango and thereby developed a high-density genetic map of mango. The characteristics and value of this genetic map were analyzed and discussed.

## Materials and methods

### Plant materials

The F_1_ mapping population consisted of 173 individuals from a cross between *M. indica* “Jin-Hwang” (female parent) and *M. indica* “Irwin” (male parent) grown at the South Subtropical Crops Research Institute, Zhanjiang, China. The Jin-Hwang mango has a large, high-quality, and greenish yellow fruit. An important trait of the Jin-Hwang mango is its resistance to anthracnose, to which it is exposed frequently during its cultivation and postharvest storage, and severely affects the development of the mango industry (Lei et al., 2006). The Irwin mango has a medium-sized, high-quality bright yellow fruit with a crimson red blush. However, it is susceptible to anthracnose (Campbell, 1992; Lei et al., 2006).

Young leaves from the parents and F_1_ individuals were collected and genomic DNA was isolated by the cetyltrimethylammonium bromide (CTAB) method (Kashkush et al., 2001). The genomic DNA was visualized by electrophoresis in agarose gel and quantified using a NanoDrop 2000 Spectrophotometer (Thermo scientific, USA).

### SLAF library construction and high-throughput sequencing

The SLAF-seq strategy (Sun et al., 2013) was used in this study. The genomic DNA of the two parents and F_1_ population was digested using *Hpy166II* restriction enzyme [New England Biolabs (NEB), USA]. Subsequently, Klenow Fragment (3′ → 5′ exo-) (NEB) and dATP were used to add a single nucleotide (A) overhang to the digested fragments at 37°C. T4 DNA ligase was used to ligate the duplex tag-labeled sequencing adapters (PAGE-purified; Life Technologies, USA) to the A-tailed fragments. PCR was carried out using diluted restriction-ligation DNA samples, Q5® High-Fidelity DNA Polymerase (NEB), dNTPs, the forward primer (5′-AATGATACGGCGACCACCGA-3′), and the reverse primer (5′-CAAGCAGAAGACGGCATACG-3′). The PCR products were then purified using Agencourt AMPure XP beads (Beckman Coulter, High Wycombe, UK) and pooled. The pooled samples were separated using 2% agarose gel electrophoresis. Fragments ranging from 264 to 464 bp (with indexes and adaptors) in size were excised and purified using a QIAquick gel extraction kit (Qiagen, Hilden, Germany). Gel-purified products were then diluted. Paired-end sequencing (125 bp from both ends) was performed using an Illumina HiSeq 2500 system (Illumina, Inc., San Diego, CA, USA) according to the manufacturer's instructions.

### Sequence data grouping and genotyping

SLAF marker identification and genotyping were performed using the procedures described by Sun et al. (2013). Low-quality reads (quality score < 20 e) were deleted and the raw reads were assigned to 173 individuals samples according to the duplex barcode sequences. After trimming the barcodes and the terminal 5-bp positions from each high-quality read, the clean reads were clustered together according to their sequence identities. Sequences mapping to the same locus with over 90% identity were defined as one SLAF locus (Zhang et al., 2015). SNP loci between the two parents were detected, and the SLAFs with >3 SNPs were removed. Alleles of each SLAF were defined according to parental reads with sequence depth >10-fold and offspring reads with sequence depth >2-fold. As *M. indica* is a diploid species, one SLAF locus can contain maximum 4 genotypes; therefore, the SLAF loci with >4 alleles were eliminated. Only SLAFs with 2, 3, or 4 alleles were found to be polymorphic and considered potential markers. All polymorphism markers were grouped into eight segregating patterns. As the map was constructed using the F_1_ population from two heterozygous parents, the markers from the segregation pattern of aa × bb were filtered out.

In order to ensure the genotyping quality, genotype scoring was conducted using a Bayesian approach as described by Sun et al. (2013). Subsequently, three steps were taken to screen the high-quality markers. First, the markers whose average sequence depths were < 10-fold in parents and < 6-fold in progeny were filtered. Second, the markers with >10% missing data were removed. Third, the markers with significant segregation distortion (*P* < 0.05) based on the chi-square test were initially excluded from the genetic map construction and then added later as accessory markers.

### Genetic map construction

Marker loci were partitioned primarily into linkage groups (LGs) by modified logarithm of odds (MLOD) scores >5. For efficient map construction, the HighMap strategy was used to arrange the SLAF markers in a specific order and correct genotyping errors within LGs (Liu et al., 2014). The genetic map was constructed according to the maximum likelihood method (van Ooijen, 2011) and the genotyping errors were corrected by the SMOOTH algorithm (van Os et al., 2005). The missing genotypes were imputed by using a k-nearest neighbor algorithm (Huang et al., 2011). The Kosambi mapping function was applied to estimate the genetic map distances in centimorgan (cM; Kosambi, 1943).

## Results

### Analysis of SLAF sequencing data and genotyping

**Figure 1:**
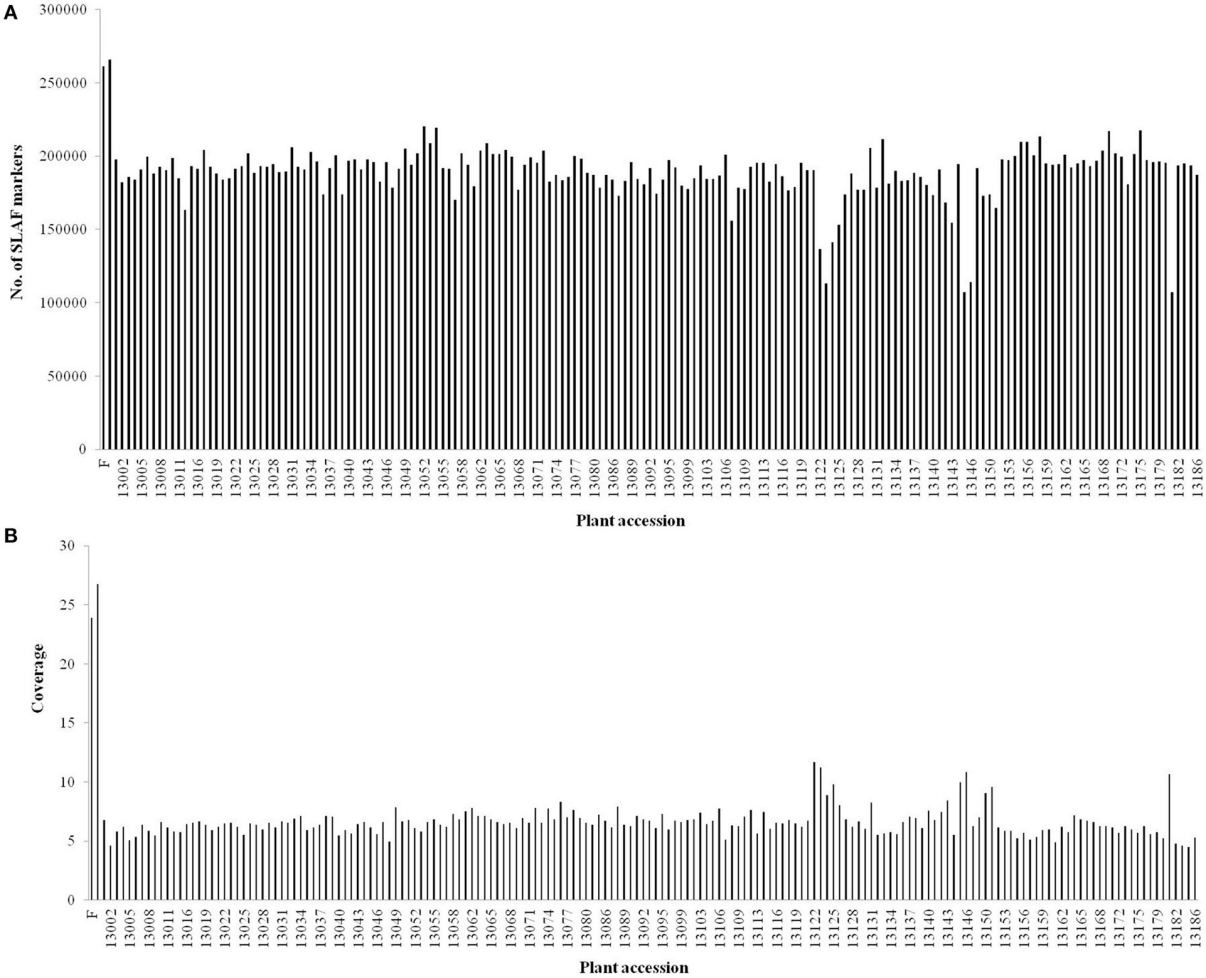
**Number and coverage of valid SLAF markers for each F_1_ individual**. The x-axis indicates the plant accession including female parent (F), male parent (M), and each of the F_1_ individuals; the y-axis indicates the number of SLAF markers **(A)** and the coverage **(B)**.

After preprocessing, 66.02 Gb of raw data containing 330.64 M reads were generated. The raw data of SLAF-seq were submitted to the Sequence Read Archive (SRA) database (accession number: SRX1741570) of the National Center of Biotechnology Information (NCBI). On an average, Q30 (quality scores of at least 30, indicating a 1% chance of error) was 85.60% and the GC content was 36.55%. The numbers of reads for female and male parents were 9,403,617 and 10,407,769, respectively. The read numbers for each F_1_ individual ranged from 1,178,049 to 2,363,336 with an average of 1,796,718 (Table S1). After read clustering, a total of 318,414 SLAFs were detected, and their average sequencing depth was found to be 25.35-fold and 6.54-fold for parents and each progeny, respectively (Figure 1).

**Figure 2:**
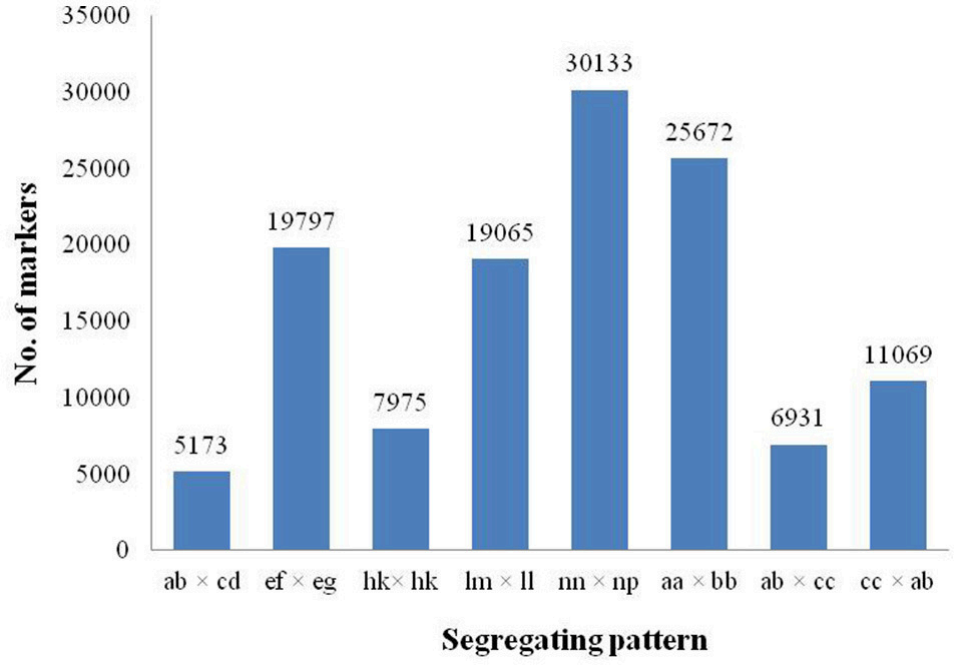
**Number of polymorphic SLAF markers for eight segregation patterns**. The x-axis indicates eight segregation patterns of polymorphic SLAF markers; the y-axis indicates the number of markers.

Among the 318,414 SLAFs, 156,368 were polymorphic with a polymorphism rate of 49.11%, while the remaining 162,046 were non-polymorphic or repetitive. After filtering the SLAF markers lacking the parent information, 125,815 polymorphic markers were successfully genotyped and grouped into eight segregation patterns (ab × cd, ef × eg, hk × hk, lm × ll, nn × np, aa × bb, ab × cc, and cc × ab; Figure 2). Since the two parents were heterozygous, the markers from the segregation pattern of aa × bb were filtered out.

### Basic characteristics of the genetic map

**Figure 3:**
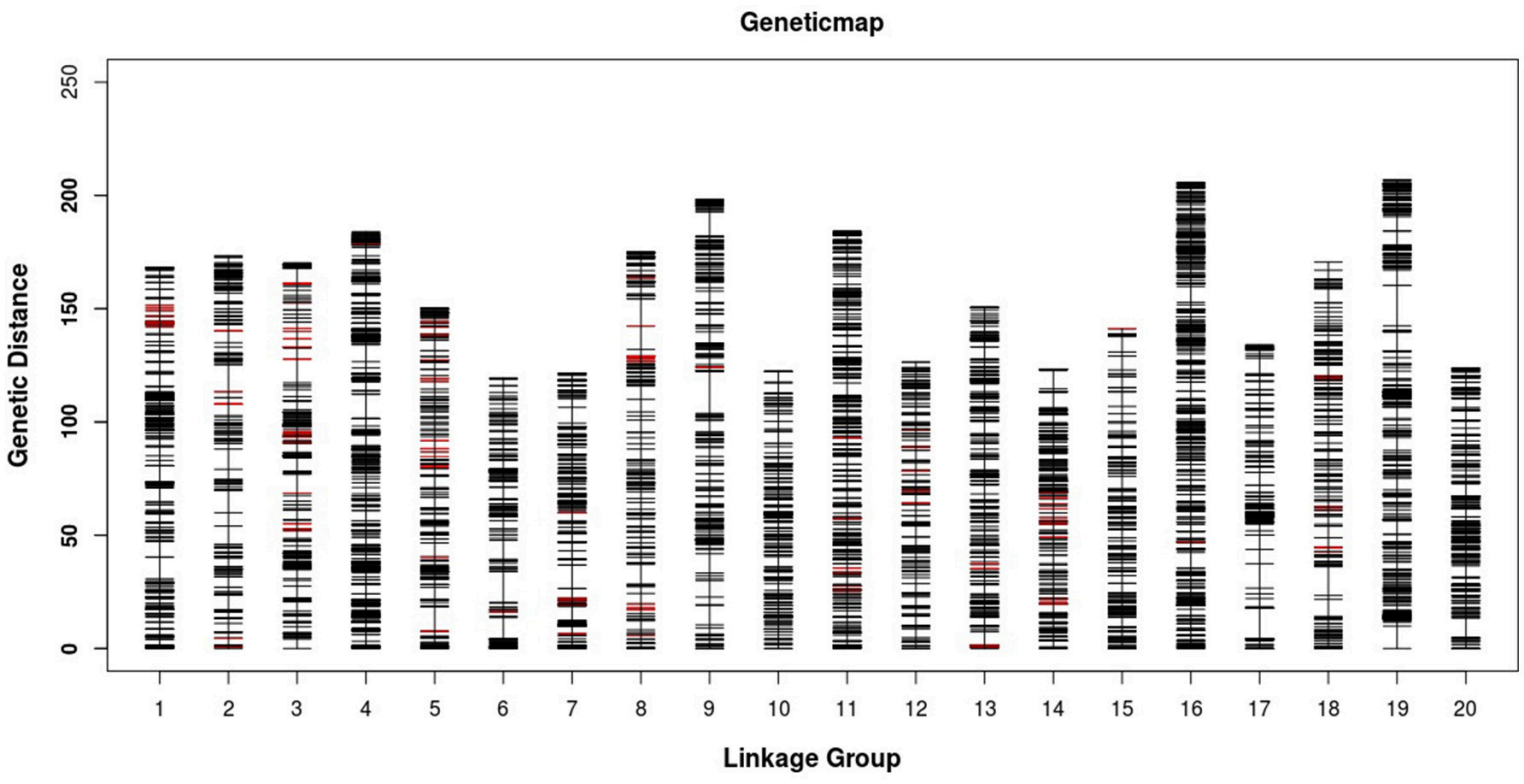
**Distribution of SLAF markers on the 20 linkage groups of mango**. A black bar indicates a SLAF marker. A red bar indicates a segregation distortion marker. The x-axis represents the linkage group number and the y-axis indicates the genetic distance (cM) in each linkage group.

After a series of screenings, 7394 SLAF markers were found to be effective and used for the final linkage analysis. The average integrity of the mapped markers was 99.90%, indicating a relatively high quality of the genetic map. After linkage analysis, among the 7394 markers mapped onto the 20 LGs, 4866 markers were used for the female map, 2585 for the male map, and 6594 for the integrated map (Figure 3, Tables S2–S4). The total genetic lengths of the female, male, and integrated maps were 3144.23, 2747.89, and 3148.28 cM, respectively. The average distances between the adjacent markers in the female, male, and integrated maps were 0.65, 1.07, and 0.48 cM, respectively.

All LGs are shown in Table 1. For the integrated map, the longest LG was LG19 (206.90 cM), which contained 520 SLAF markers, while the shortest LG (LG6, 119.44 cM) harbored 255 SLAF markers. LG16 had the maximum markers (591), whereas LG8 possessed the minimum markers (219). On an average, each LG contained 330 SLAF markers. The genetic length of the LGs ranged from 119.44 cM (LG6) to 206.90 cM (LG19), with an average distance between the adjacent markers ranging from 0.35 cM (LG16) to 0.8 cM (LG8). The “Gap ≤ 5” value, which reflected the degree of linkage among markers, ranged from 95.15 to 99.49%, with the largest gap of 18.734 cM located in LG9. A total of 13,844 SNP loci were identified among the 6594 mapped SLAF markers (Table 2). LG16 had the maximum SNP loci (1139), whereas LG8 contained the minimum SNPs (443).

**Table 1:** **Basic characteristics of the 20 linkage groups of mango**.

**Linkage group**	**Total number of markers**			**Total distance (cM)**			**Average distance (cM)**			**Gap<5 (cM)**			**Max gap (cM)**			**Number of markers with segregation distortion**
	**Female map**	**Male map**	**Integrated map**	**Female map**	**Male map**	**Integrated map**	**Female map**	**Male map**	**Integrated map**	**Female map**	**Male map**	**Integrated map**	**Female map**	**Male map**	**Integrated map**	**Integrated map**
LG1	230	145	362	161.96	113.72	168.04	0.71	0.79	0.47	96.51%	92.36%	98.06%	22.148	22.188	9.612	17
LG2	170	111	272	180.35	165.48	173.21	1.07	1.50	0.64	94.67%	93.64%	97.05%	21.295	23.033	12.019	8
LG3	245	67	299	198.74	104.87	170.13	0.81	1.59	0.57	96.31%	90.91%	98.32%	13.030	14.758	10.450	28
LG4	390	162	526	188.39	137.92	184.04	0.48	0.86	0.35	99.23%	95.65%	99.43%	13.548	31.201	10.715	1
LG5	209	80	262	144.75	88.97	150.23	0.70	1.13	0.58	95.67%	93.67%	97.70%	10.94	17.055	10.940	22
LG6	207	76	255	146.29	108.31	119.44	0.71	1.44	0.47	98.06%	92.00%	96.85%	16.249	38.316	13.690	3
LG7	195	49	224	125.31	116.33	121.40	0.65	2.42	0.54	96.39%	91.67%	97.76%	12.290	39.192	10.311	9
LG8	191	74	219	167.78	105.03	175.09	0.88	1.44	0.80	97.37%	91.78%	96.79%	28.000	45.865	12.075	15
LG9	202	171	368	182.83	258.06	198.19	0.91	1.52	0.54	95.02%	93.53%	97.28%	20.624	45.959	18.734	3
LG10	192	130	287	119.61	122.7	122.33	0.63	0.95	0.43	96.86%	96.12%	99.30%	9.61	34.369	5.144	0
LG11	392	235	480	178.46	189.62	184.04	0.46	0.81	0.38	98.21%	96.58%	99.37%	9.523	19.466	5.388	12
LG12	160	108	231	136.15	106.6	126.40	0.86	1.00	0.55	93.71%	93.46%	98.70%	12.401	21.295	5.245	9
LG13	264	160	371	160.1	141.03	150.57	0.61	0.89	0.41	97.72%	93.08%	98.92%	16.249	21.295	6.088	17
LG14	219	86	272	116.84	109.00	123.21	0.54	1.28	0.45	96.33%	96.47%	98.89%	6.143	46.874	8.047	14
LG15	183	98	262	140.72	85.10	141.19	0.77	0.88	0.54	95.05%	92.78%	96.17%	9.926	16.249	6.801	4
LG16	477	223	591	206.09	169.13	205.69	0.43	0.76	0.35	98.74%	96.40%	99.49%	11.665	31.035	6.967	1
LG17	174	80	228	132.75	116.64	133.93	0.77	1.48	0.59	95.38%	86.08%	95.15%	45.959	24.032	13.799	0
LG18	158	160	266	140.96	192.00	170.57	0.90	1.21	0.64	93.63%	96.23%	98.11%	21.026	33.952	12.696	11
LG19	359	294	520	193.2	258.51	206.90	0.54	0.88	0.40	98.04%	95.22%	98.27%	38.932	27.908	17.809	0
LG20	249	76	299	122.95	58.87	123.68	0.50	0.78	0.42	97.18%	88.00%	97.99%	10.227	6.705	8.827	0
Total	4866	2585	6594	3144.23	2747.89	3148.28	0.65	1.07	0.48	96.50%	93.28%	97.98%	45.959	46.874	18.734	174

“*Gap < 5” indicates the percentages of gaps in which the distance between the adjacent markers was less than 5 cM*.

**Table 2:** **Distribution of SNP loci on the 20 linkage groups of mango**.

**Linkage group**	**SNP number**	**Transition/Transversion number**
LG1	750	487/263
LG2	575	371/204
LG3	691	439/252
LG4	1112	692/421
LG5	592	404/189
LG6	561	389/172
LG7	512	340/172
LG8	443	278/165
LG9	775	497/279
LG10	596	421/176
LG11	971	578/393
LG12	474	312/162
LG13	788	523/265
LG14	562	355/207
LG15	506	319/188
LG16	1139	726/414
LG17	515	351/164
LG18	549	329/221
LG19	1105	705/400
LG20	628	397/231

### Quality evaluation of the genetic map

Quality of the mango genetic map was evaluated by constructing the haplotype and heat maps. The haplotype maps, which reflect the double exchange of the population, were developed for the parental controls and 173 offsprings using 6594 SLAF markers (Supplementary Material Presentation 1). Most of the recombination blocks were distinctly defined. The missing data for each LG ranged from 0.04% (LG1) to 0.22% (LG8). Most of the LGs were uniformly distributed, suggesting that the genetic maps were of high quality.

The heat maps showed the relationships of recombination between markers from each LG. Pair-wise comparisons between markers were used to assign recombination scores to 6594 markers, after which the heat maps were constructed (Supplementary Material Presentation 2). The resulting maps showed that the order of SLAF markers in most of the LGs was correct.

### Segregation distortion markers on the map

Of the 6594 markers, only 174 (2.64%) exhibited significant segregation distortion (*P* < 0.05) on the genetic map on the basis of a chi-square test (Table 1). The segregation distortion markers were distributed on most of the LGs with the exceptions of LG10, LG17, LG19, and LG20. The frequencies of distorted markers on LG3 (16.09%) and LG5 (12.64%) were higher than those of the other LGs. No significant correlation was observed between the distribution of the distorted and mapped markers. For example, LG16, which possessed the maximum markers (591 SLAF markers) and covered 205.69 cM, included only one distorted marker. Comparatively, LG8, which possessed the minimum markers (219 SLAF markers), included 15 distorted markers.

## Discussion

The development of abundant and reliable molecular markers was very important for the construction of a genetic map. In the present study, SNP markers were used for map construction. SLAF-seq technology is an efficient method of *de novo* SNP detection and large-scale genotyping, which is based on RRL and high-throughput sequencing (Sun et al., 2013). The markers developed using SLAF-seq technology were of relatively higher density, better consistency and effectiveness, and lower cost than those developed using traditional methods. Since the development of SLAF-seq technology, it has been applied in many plant studies and has produced remarkable results. Zhang et al. (2013) employed SLAF-seq technology to detect 71,793 high-quality SLAFs, of which 3673 were polymorphic; they used 1233 of the polymorphic markers to construct the first high-density genetic map for sesame. Qi et al. (2014) applied SLAF-seq to develop 12,577 polymorphic SLAFs, and used 5308 of them to construct a soybean genetic map. Similar studies have been conducted on many other crops, such as wax gourd (22,151 polymorphic SLAFs; Jiang et al., 2015), tea plant (25,014 polymorphic SLAFs; Ma et al., 2015), walnut (49,174 polymorphic SLAFs; Zhu et al., 2015), mei (93,031 polymorphic SLAFs; Zhang et al., 2015), grapevine (42,279 polymorphic SLAFs; Guo et al., 2015), red sage (62,834 polymorphic SLAFs; Liu et al., 2016), and cucumber (15,946 polymorphic SLAFs; Zhu et al., 2016). In this study, 66.02 Gb of raw data containing 330.64 M reads were generated through SLAF-seq. There were a total of 318,414 SLAF markers, of which 156,368 were polymorphic. After a series of screenings, 7394 SLAF markers were found to be effective and were used for linkage analysis; however, only 6594 markers containing 13,844 SNP loci were mapped successfully onto the high-density genetic map. The average sequencing depth of the markers that failed to be included in the map was 535,794, and the average integrity of all samples was 95%, which suggested that this failure was not due to a sequencing problem. The polymorphic markers of mango developed by SLAF-seq technology were more numerous than those of the previously mentioned crops, further demonstrating the potential of SLAF-seq as a low-cost technique to effectively develop numerous and reliable molecular markers for fruit trees.

Genetic maps are the basis of QTL analyses of the agronomic traits, which are important for the improvement of breeding programs. However, the construction of genetic maps is difficult, and there is a shortage of genetic maps for fruit trees, especially the tropical ones. There were a few reports for papaya (Sondur et al., 1996), avocado (Sharon et al., 1997), mango (Chunwongse et al., 2000, 2015; Kashkush et al., 2001; Fang et al., 2003), and guava (Padmakar et al., 2015) with respect to genetic maps. Although four genetic maps have been previously reported for mango, there were several deficiencies in them. Firstly, the maximum number of F_1_ individuals used for these studies was 60. If the size of the population used for map construction is too small, it cannot reflect small recombination rates, and is not beneficial for increasing the efficiency of QTL detection. Secondly, the dominant molecular markers, such as AFLP, were mainly employed, whereas the co-dominant markers, such as SSR, were rarely used. Thirdly, the number of mapped markers was relatively small, which resulted in relatively large average distances between the adjacent markers. The progress of the genetic map construction was very slow, and to the best of our knowledge, no high-density map has been reported to date. The total length of the new genetic map, which was constructed using 6594 SLAF markers in this study, was 3148.28 cM spanning 20 LGs, and the average distance between adjacent markers was 0.48 cM. This map could be considered the first high-density genetic map of mango, and its evaluation using haplotype and heat maps suggested that it was of high quality (Supplementary Material Presentations 1, 2).

Some of the seedling trees of the F_1_ progenies of the two cultivars of mango began to set fruit two years ago, and the phenotypic data of the two parents and F_1_ progenies were collected. The characteristic of disease resistance was segregated. Since the Jin-Hwang mango is resistant to anthracnose and Irwin mango is susceptible to anthracnose, the cross between the two cultivars might be used to breed a new variety with high anthracnose resistance and enable the study of the inheritance of resistance. The high-density genetic map of mango constructed here will be applied to map the QTL/QTLs of this trait.

Additionally, our sequencing results provided a mass of SLAF markers for mango. Because they were explored at the whole-genome level, the sequence and location information of these markers can be used as a reference in the future genome assemblies of mango. Furthermore, a total of 13,844 SNPs, which are co-dominant markers, were found among the SLAF markers, and they can be used for comparative genomic studies and future MAS in mango breeding program. Like other molecular markers, the SLAFs and SNPs developed here also can be applied to other mango cultivars and progenies for identification of the germplasm or hybrid and analysis of the genetic diversity among cultivars.

## Author contributions

Conceived and designed the experiments: CL, BS, and QY. Performed the experiments and analyzed the data: CL, BS, and HW. Wrote the manuscript: CL, BS. Read and approved the final manuscript: WX, SW.

### Conflict of interest statement

The authors declare that the research was conducted in the absence of any commercial or financial relationships that could be construed as a potential conflict of interest.

## Supplementary material

Supplementary Material Presentation 1 **Haplotype map of the integrated maps**. Each row represents a marker. Markers are ranked in accordance with the map order. Each of the two columns represents an individual; blank columns are used between two individuals. The first and second columns represent the paternal and maternal chromosomes, respectively. The green and blue areas in the columns represent the first and second alleles from the parents, respectively. The white column represents the source of alleles that cannot be judged. The gray areas represent the deleted alleles.

Supplementary Material Presentation 2 **Heat map of the integrated maps**. Markers of each row and column are ranked according to the map order; each small square represents the rate of recombination (r) between the two markers.

Table S1 **Summary of the sequencing data**.

Table S2 **SLAF markers and SNP loci on the map**. The SNPs within the SLAF marker allele sequences were marked in lowercase letters. The SLAF markers separated by linkage groups are shown in different sheets and the parent from which the alleles were derived are shown.

Table S3 **Details of marker genotypes for F1 individuals**.

Table S4 **SLAF markers and their locations in each LG on the map**.
