# Supplementary figures and images for: Construction of a High-Density Genetic Map Based on Large-Scale Marker Development in Mango Using Specific-Locus Amplified Fragment Sequencing (SLAF-seq)
Source: Front Plant Sci. 2016 Aug 30;7:1310. doi: 10.3389/fpls.2016.01310 (PMC5003885; doi:10.3389/fpls.2016.01310)

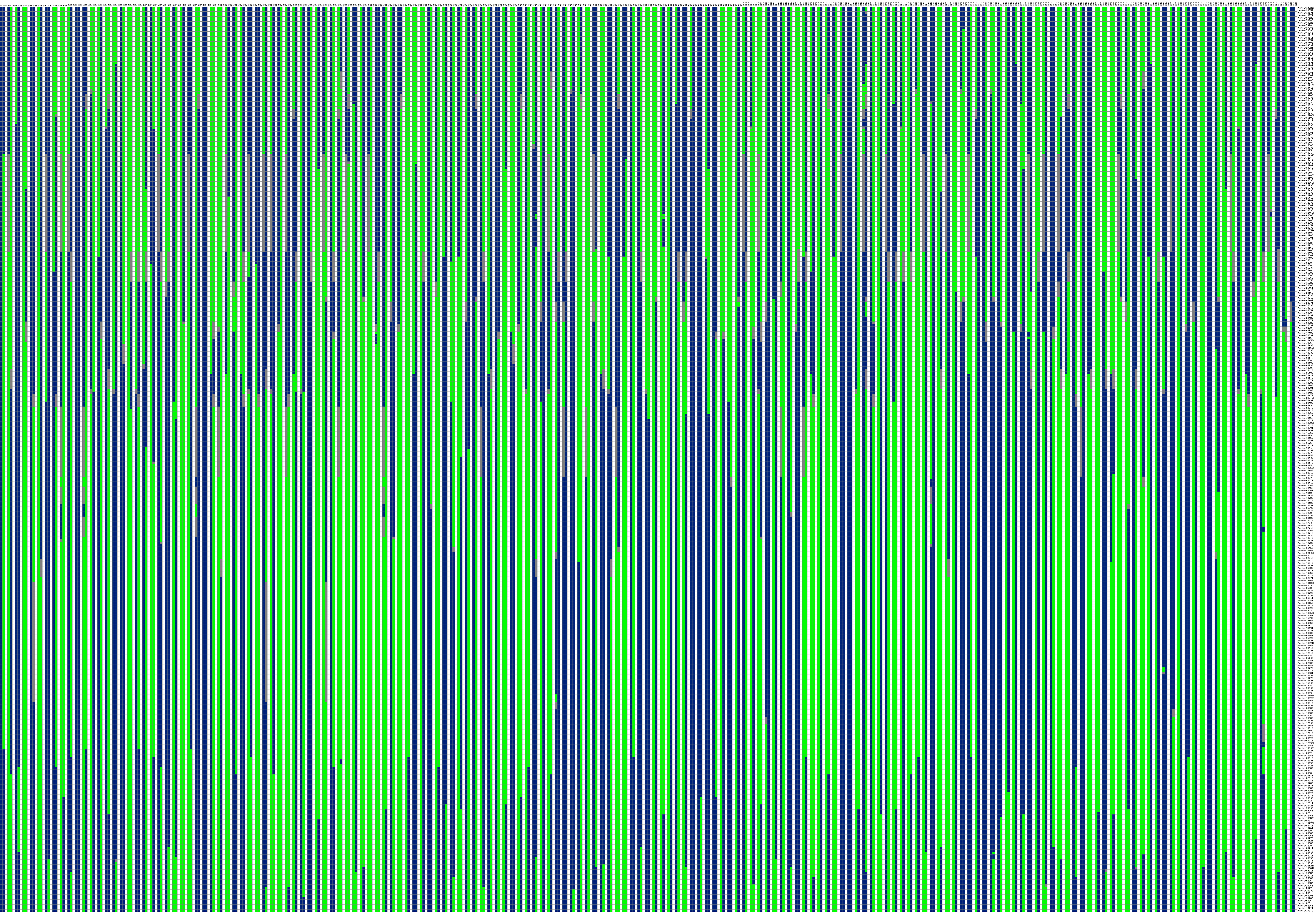

Supplement: Supplementary Material Presentation 1 — Haplotype map of the integrated maps. Each row represents a marker. Markers are ranked in accordance with the map order. Each of the two columns represents an individual; blank columns are used between two individuals. The first and second columns represent the paternal and maternal chromosomes, respectively. The green and blue areas in the columns represent the first and second alleles from the parents, respectively. The white column represents the source of alleles that cannot be judged. The gray areas represent the deleted alleles. [file Presentation1.ZIP › Supplementary Material Presentation 1/LG1.haploMap.png]

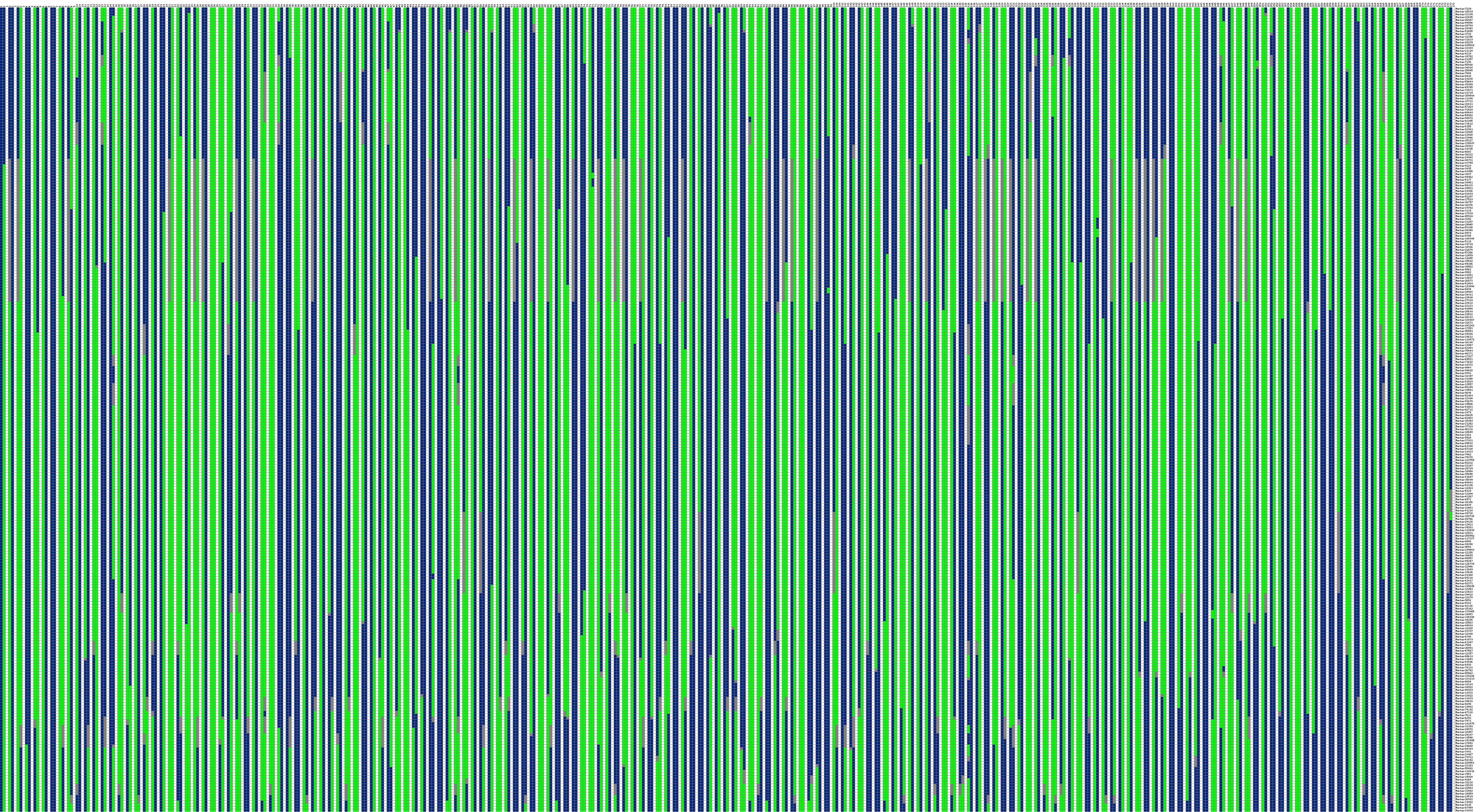

Supplement: Supplementary Material Presentation 1 — Haplotype map of the integrated maps. Each row represents a marker. Markers are ranked in accordance with the map order. Each of the two columns represents an individual; blank columns are used between two individuals. The first and second columns represent the paternal and maternal chromosomes, respectively. The green and blue areas in the columns represent the first and second alleles from the parents, respectively. The white column represents the source of alleles that cannot be judged. The gray areas represent the deleted alleles. [file Presentation1.ZIP › Supplementary Material Presentation 1/LG10.haploMap.png]

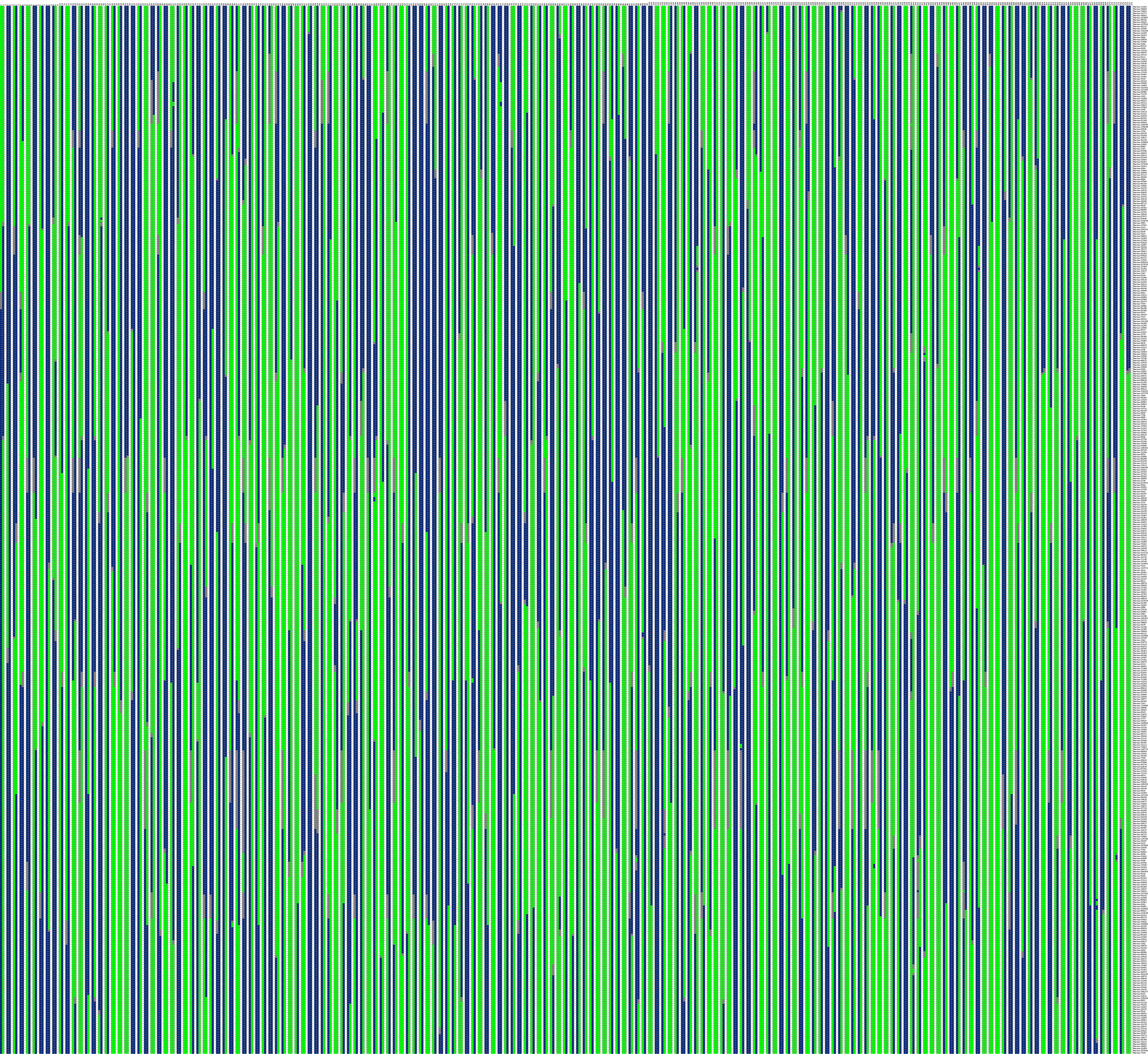

Supplement: Supplementary Material Presentation 1 — Haplotype map of the integrated maps. Each row represents a marker. Markers are ranked in accordance with the map order. Each of the two columns represents an individual; blank columns are used between two individuals. The first and second columns represent the paternal and maternal chromosomes, respectively. The green and blue areas in the columns represent the first and second alleles from the parents, respectively. The white column represents the source of alleles that cannot be judged. The gray areas represent the deleted alleles. [file Presentation1.ZIP › Supplementary Material Presentation 1/LG11.haploMap.png]

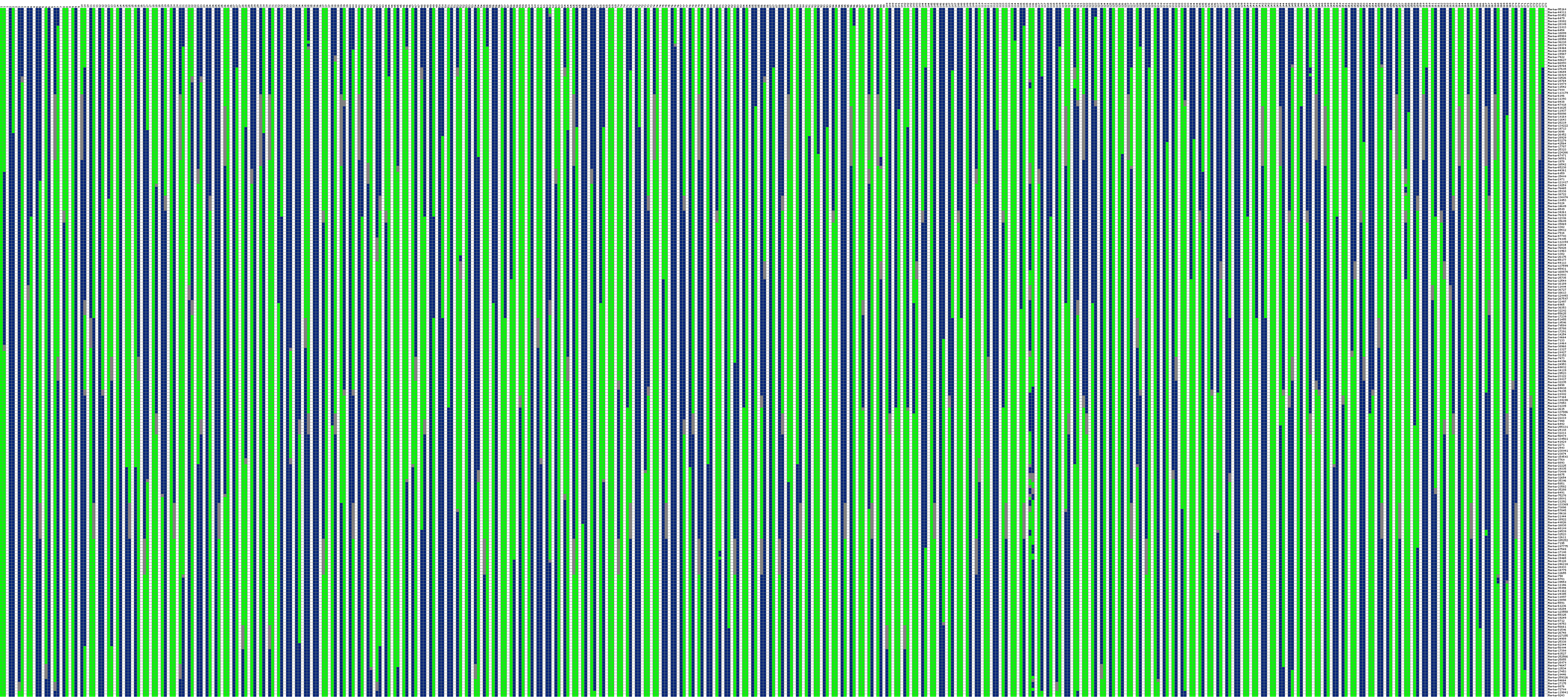

Supplement: Supplementary Material Presentation 1 — Haplotype map of the integrated maps. Each row represents a marker. Markers are ranked in accordance with the map order. Each of the two columns represents an individual; blank columns are used between two individuals. The first and second columns represent the paternal and maternal chromosomes, respectively. The green and blue areas in the columns represent the first and second alleles from the parents, respectively. The white column represents the source of alleles that cannot be judged. The gray areas represent the deleted alleles. [file Presentation1.ZIP › Supplementary Material Presentation 1/LG12.haploMap.png]

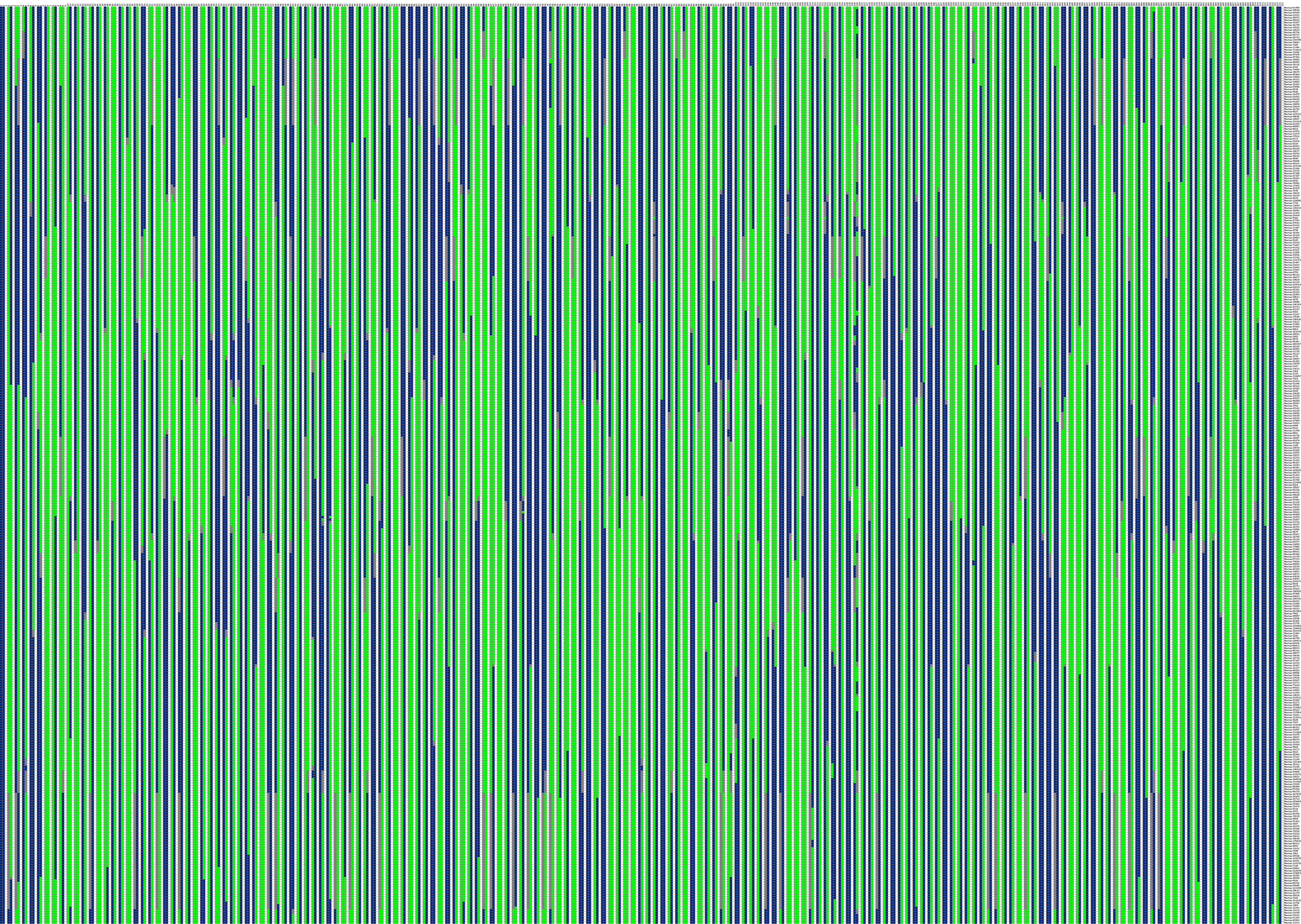

Supplement: Supplementary Material Presentation 1 — Haplotype map of the integrated maps. Each row represents a marker. Markers are ranked in accordance with the map order. Each of the two columns represents an individual; blank columns are used between two individuals. The first and second columns represent the paternal and maternal chromosomes, respectively. The green and blue areas in the columns represent the first and second alleles from the parents, respectively. The white column represents the source of alleles that cannot be judged. The gray areas represent the deleted alleles. [file Presentation1.ZIP › Supplementary Material Presentation 1/LG13.haploMap.png]

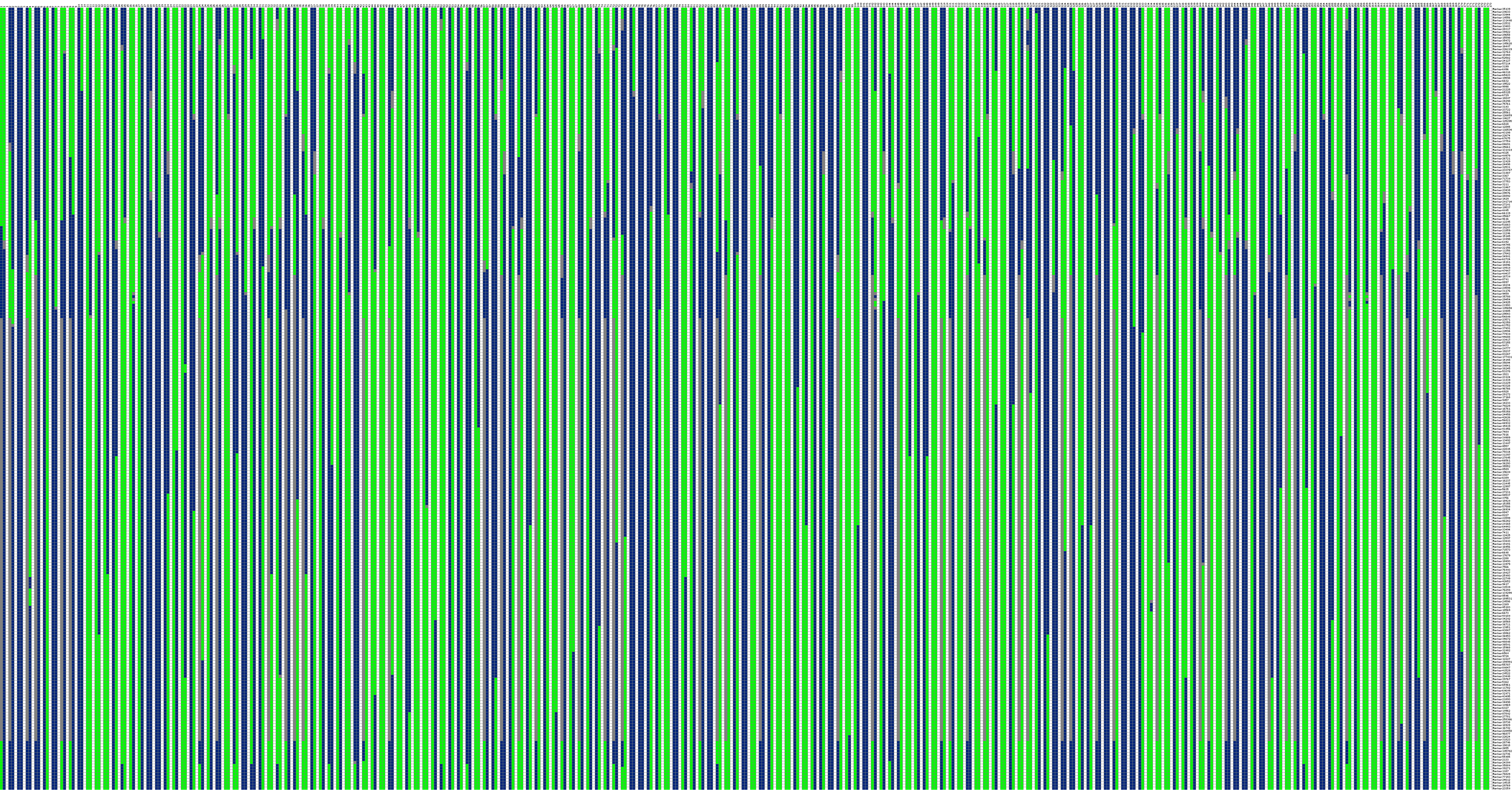

Supplement: Supplementary Material Presentation 1 — Haplotype map of the integrated maps. Each row represents a marker. Markers are ranked in accordance with the map order. Each of the two columns represents an individual; blank columns are used between two individuals. The first and second columns represent the paternal and maternal chromosomes, respectively. The green and blue areas in the columns represent the first and second alleles from the parents, respectively. The white column represents the source of alleles that cannot be judged. The gray areas represent the deleted alleles. [file Presentation1.ZIP › Supplementary Material Presentation 1/LG14.haploMap.png]

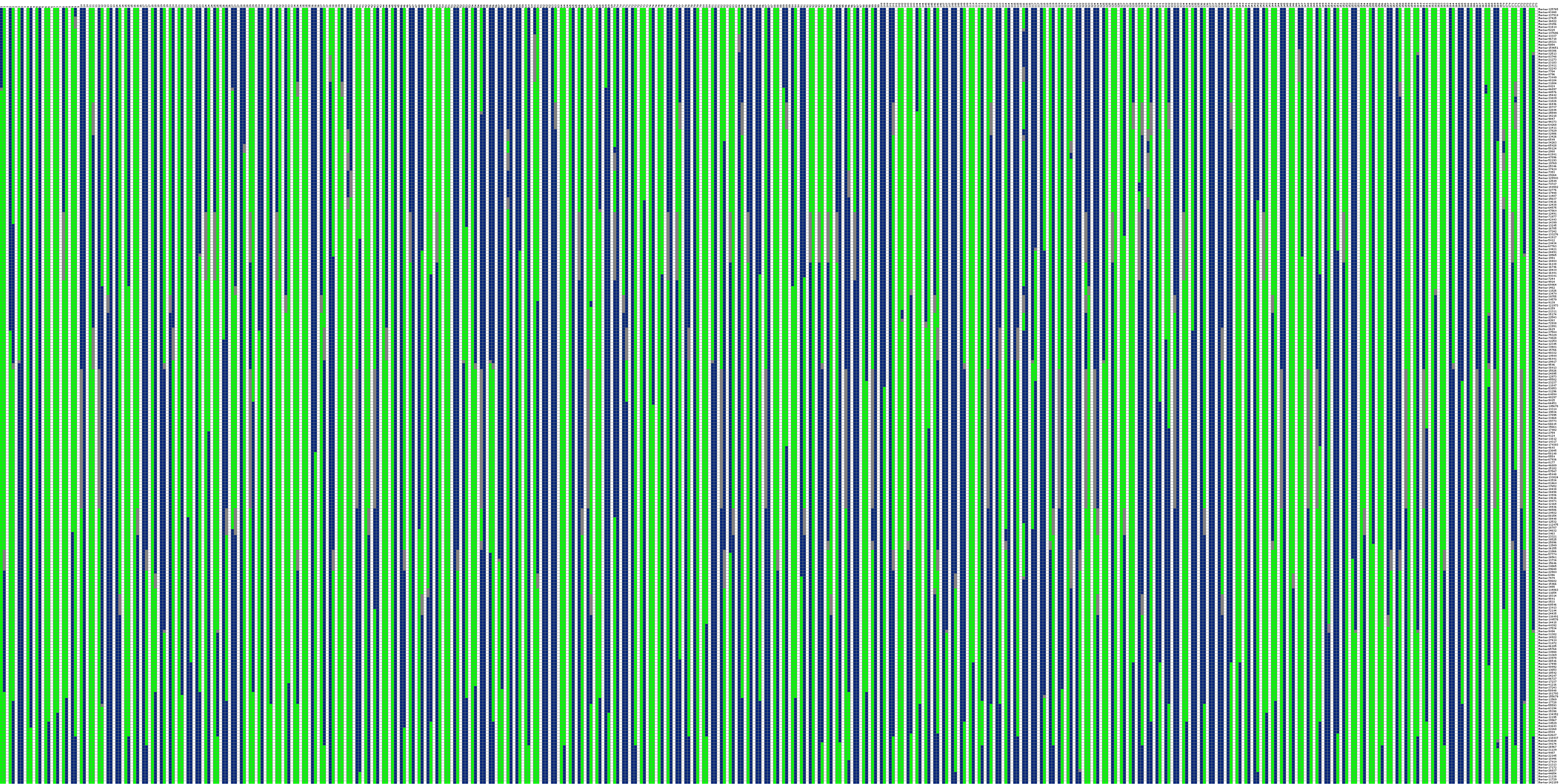

Supplement: Supplementary Material Presentation 1 — Haplotype map of the integrated maps. Each row represents a marker. Markers are ranked in accordance with the map order. Each of the two columns represents an individual; blank columns are used between two individuals. The first and second columns represent the paternal and maternal chromosomes, respectively. The green and blue areas in the columns represent the first and second alleles from the parents, respectively. The white column represents the source of alleles that cannot be judged. The gray areas represent the deleted alleles. [file Presentation1.ZIP › Supplementary Material Presentation 1/LG15.haploMap.png]

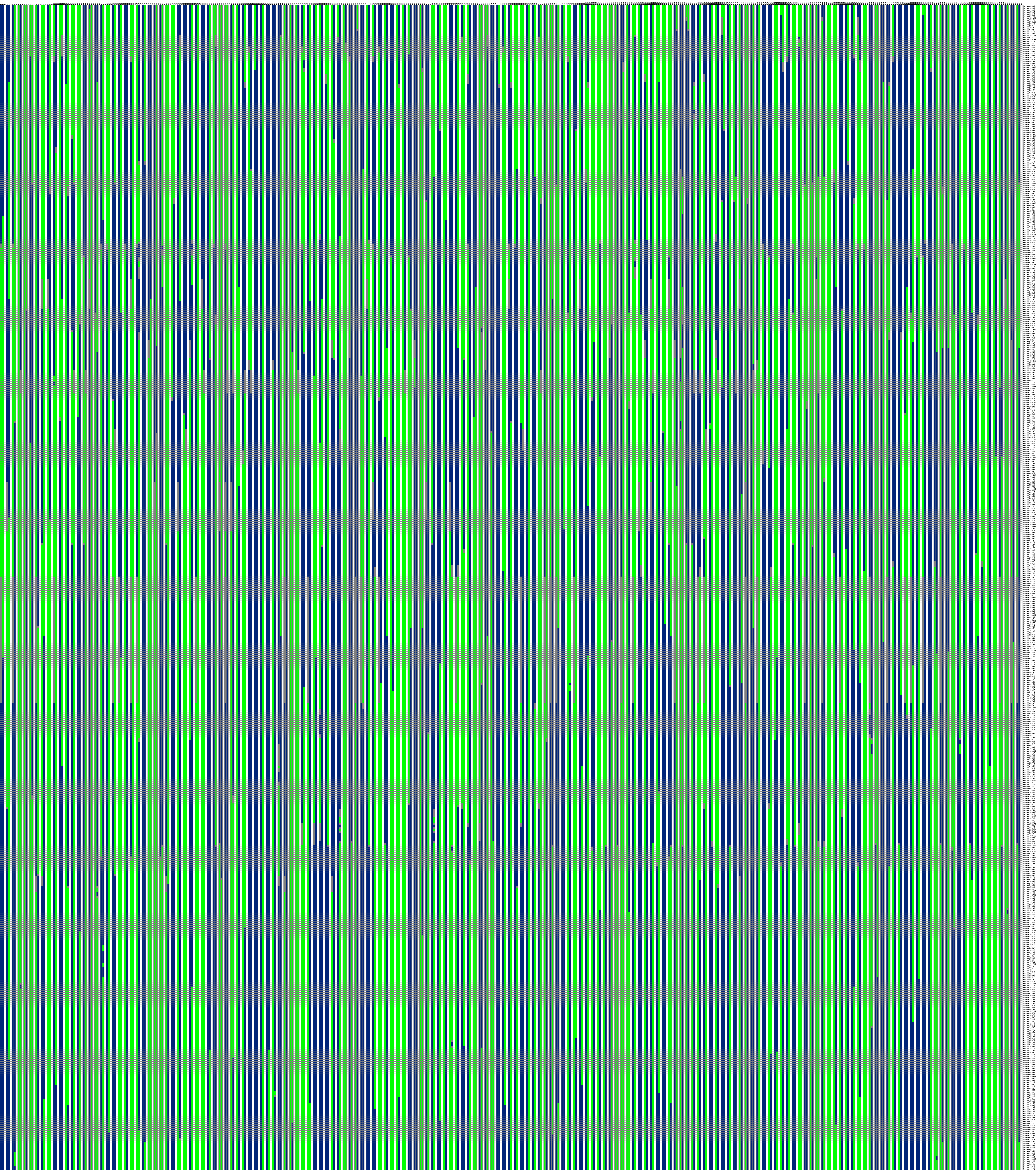

Supplement: Supplementary Material Presentation 1 — Haplotype map of the integrated maps. Each row represents a marker. Markers are ranked in accordance with the map order. Each of the two columns represents an individual; blank columns are used between two individuals. The first and second columns represent the paternal and maternal chromosomes, respectively. The green and blue areas in the columns represent the first and second alleles from the parents, respectively. The white column represents the source of alleles that cannot be judged. The gray areas represent the deleted alleles. [file Presentation1.ZIP › Supplementary Material Presentation 1/LG16.haploMap.png]

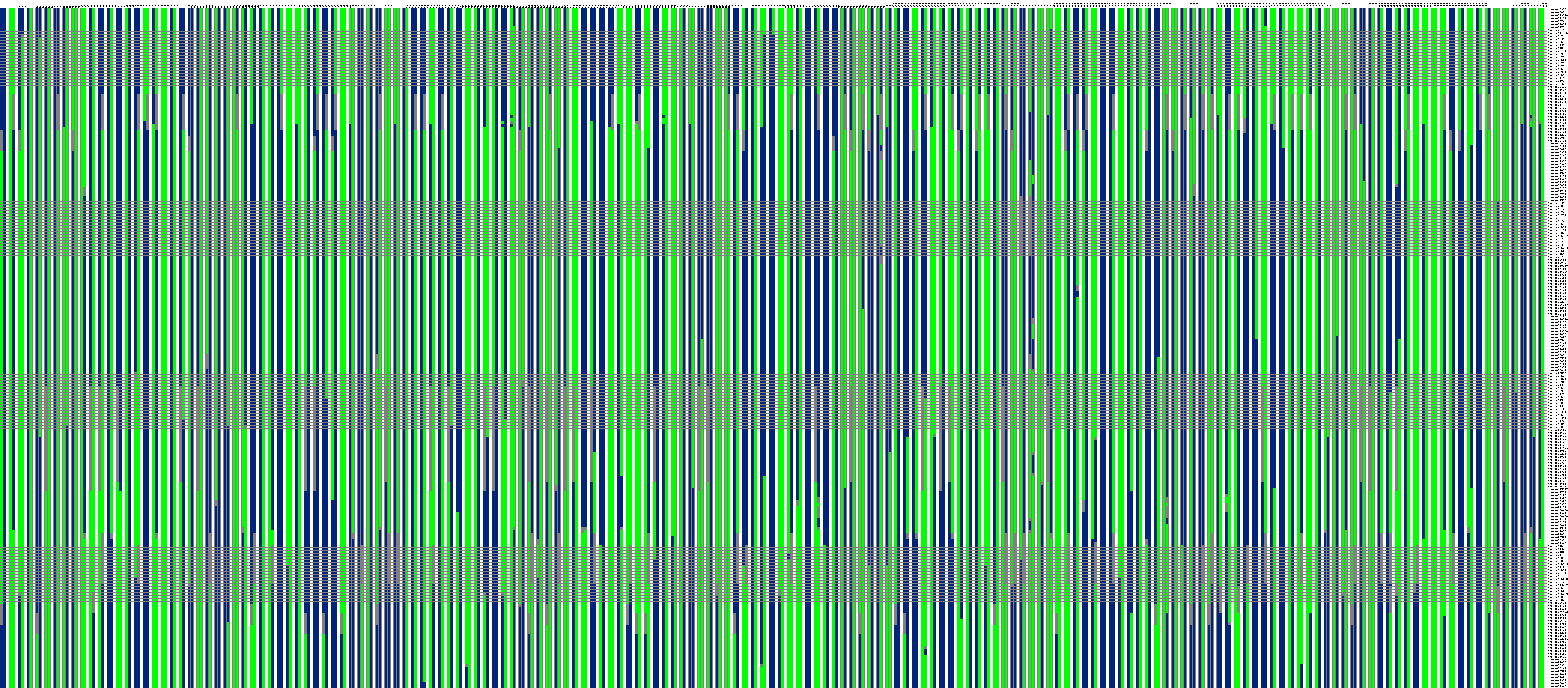

Supplement: Supplementary Material Presentation 1 — Haplotype map of the integrated maps. Each row represents a marker. Markers are ranked in accordance with the map order. Each of the two columns represents an individual; blank columns are used between two individuals. The first and second columns represent the paternal and maternal chromosomes, respectively. The green and blue areas in the columns represent the first and second alleles from the parents, respectively. The white column represents the source of alleles that cannot be judged. The gray areas represent the deleted alleles. [file Presentation1.ZIP › Supplementary Material Presentation 1/LG17.haploMap.png]

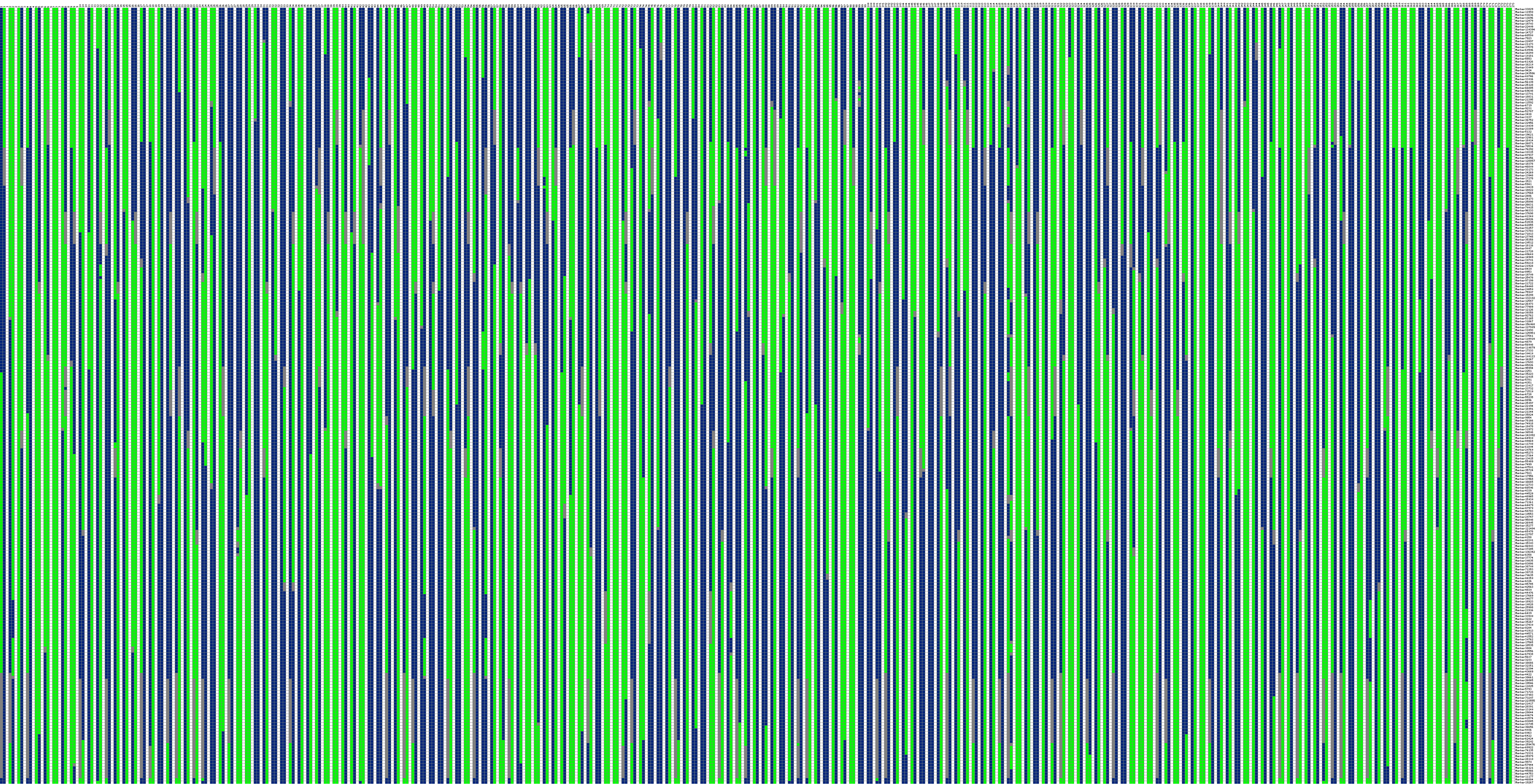

Supplement: Supplementary Material Presentation 1 — Haplotype map of the integrated maps. Each row represents a marker. Markers are ranked in accordance with the map order. Each of the two columns represents an individual; blank columns are used between two individuals. The first and second columns represent the paternal and maternal chromosomes, respectively. The green and blue areas in the columns represent the first and second alleles from the parents, respectively. The white column represents the source of alleles that cannot be judged. The gray areas represent the deleted alleles. [file Presentation1.ZIP › Supplementary Material Presentation 1/LG18.haploMap.png]

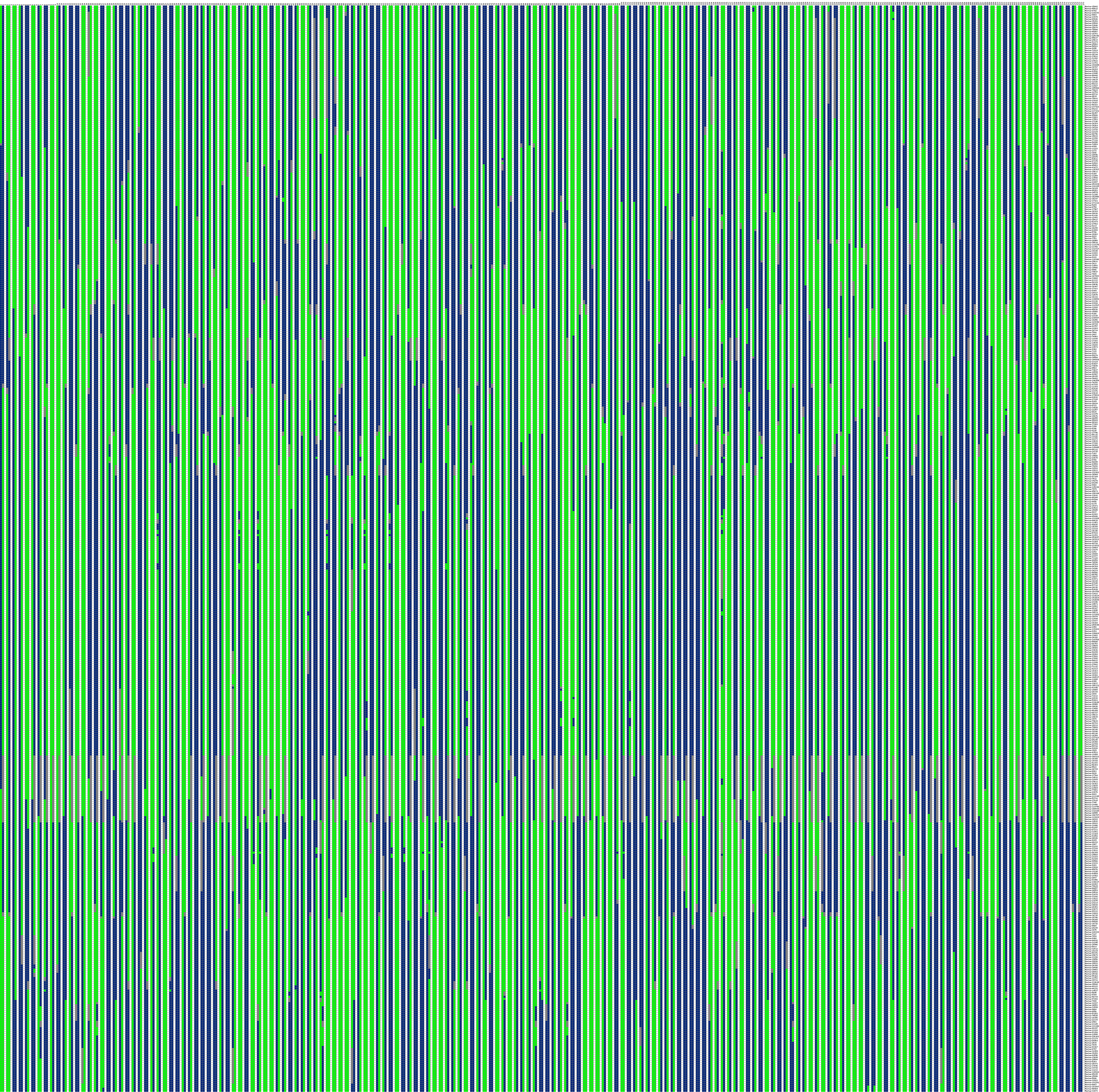

Supplement: Supplementary Material Presentation 1 — Haplotype map of the integrated maps. Each row represents a marker. Markers are ranked in accordance with the map order. Each of the two columns represents an individual; blank columns are used between two individuals. The first and second columns represent the paternal and maternal chromosomes, respectively. The green and blue areas in the columns represent the first and second alleles from the parents, respectively. The white column represents the source of alleles that cannot be judged. The gray areas represent the deleted alleles. [file Presentation1.ZIP › Supplementary Material Presentation 1/LG19.haploMap.png]

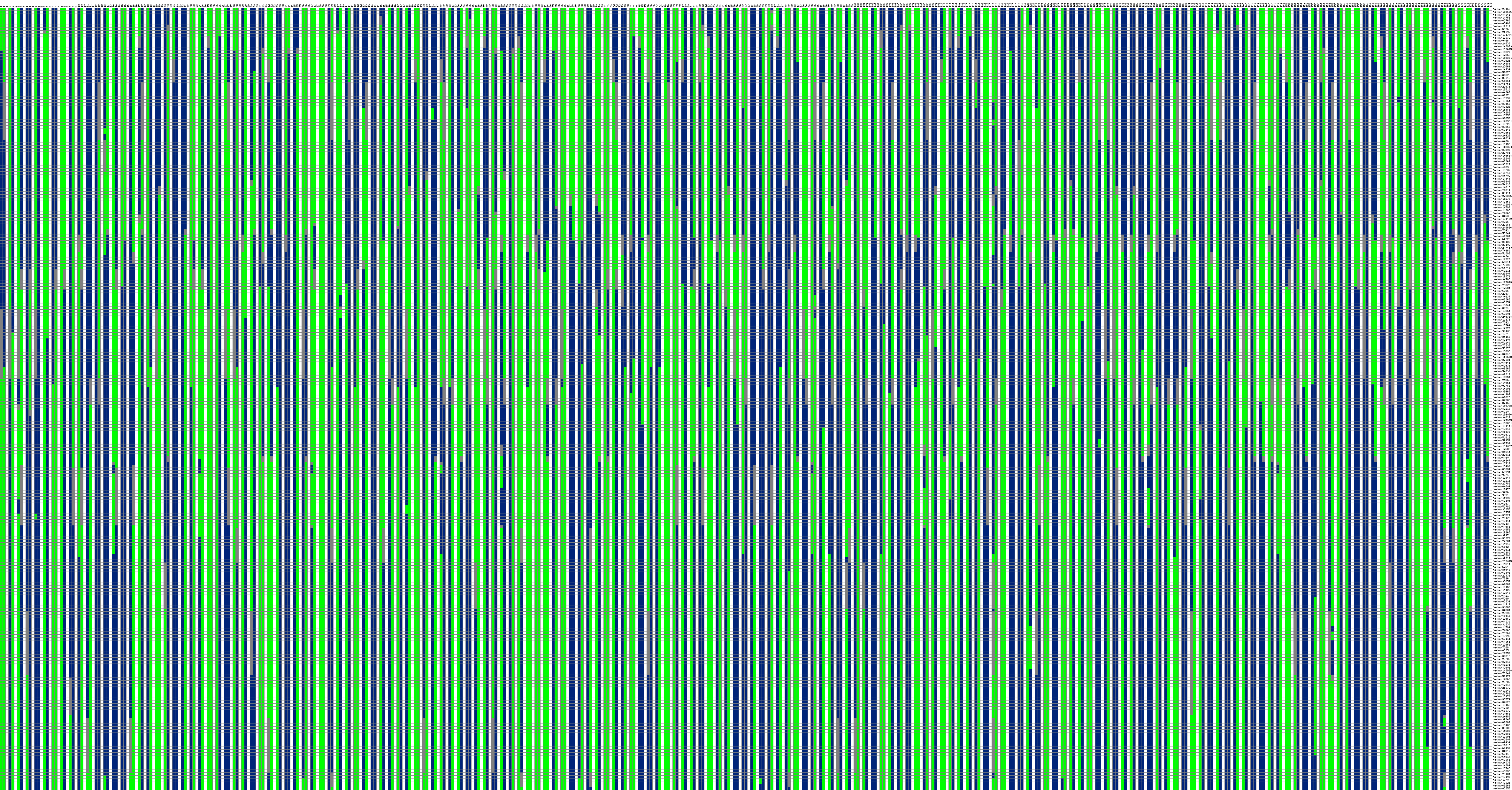

Supplement: Supplementary Material Presentation 1 — Haplotype map of the integrated maps. Each row represents a marker. Markers are ranked in accordance with the map order. Each of the two columns represents an individual; blank columns are used between two individuals. The first and second columns represent the paternal and maternal chromosomes, respectively. The green and blue areas in the columns represent the first and second alleles from the parents, respectively. The white column represents the source of alleles that cannot be judged. The gray areas represent the deleted alleles. [file Presentation1.ZIP › Supplementary Material Presentation 1/LG2.haploMap.png]

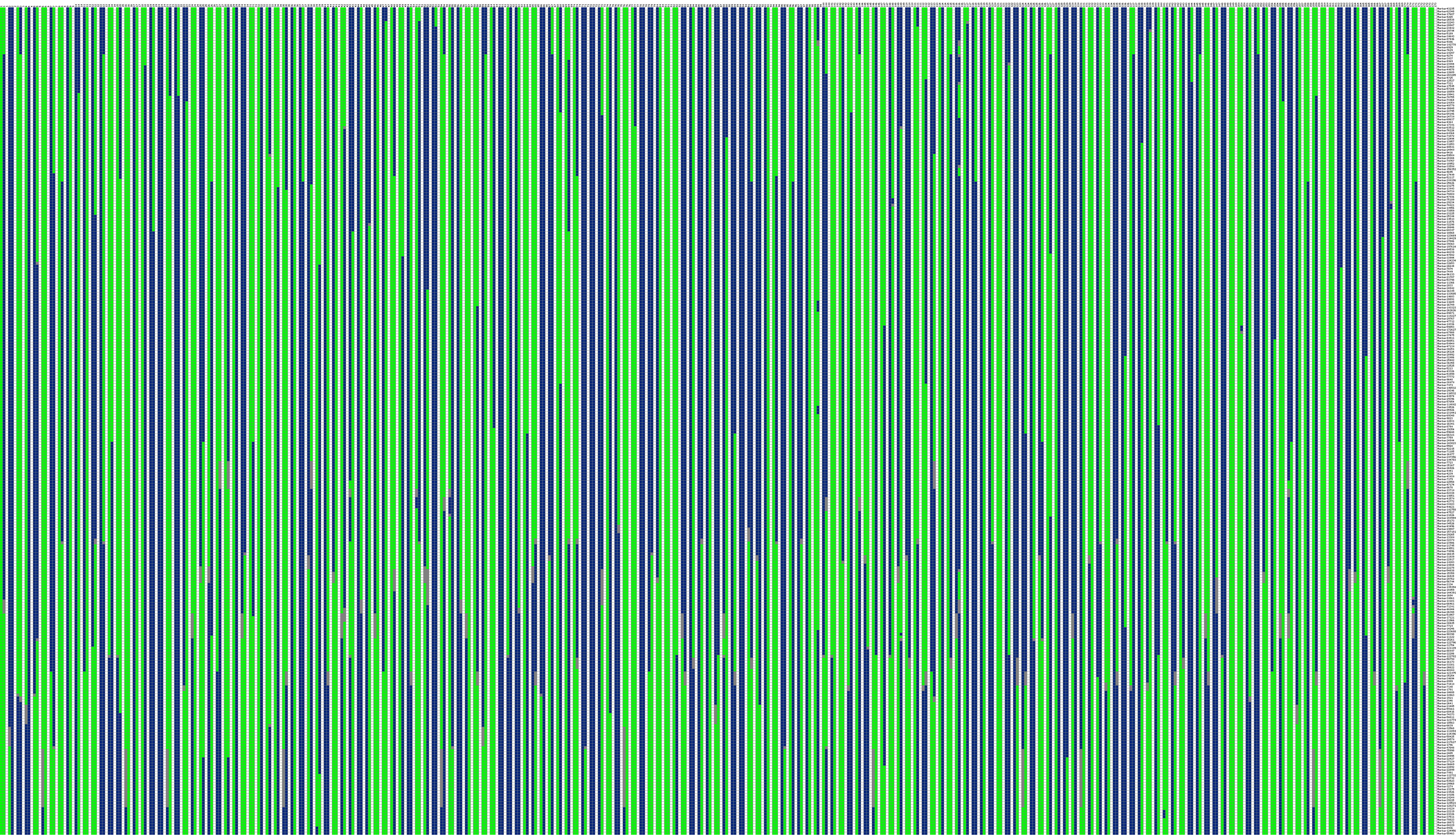

Supplement: Supplementary Material Presentation 1 — Haplotype map of the integrated maps. Each row represents a marker. Markers are ranked in accordance with the map order. Each of the two columns represents an individual; blank columns are used between two individuals. The first and second columns represent the paternal and maternal chromosomes, respectively. The green and blue areas in the columns represent the first and second alleles from the parents, respectively. The white column represents the source of alleles that cannot be judged. The gray areas represent the deleted alleles. [file Presentation1.ZIP › Supplementary Material Presentation 1/LG20.haploMap.png]

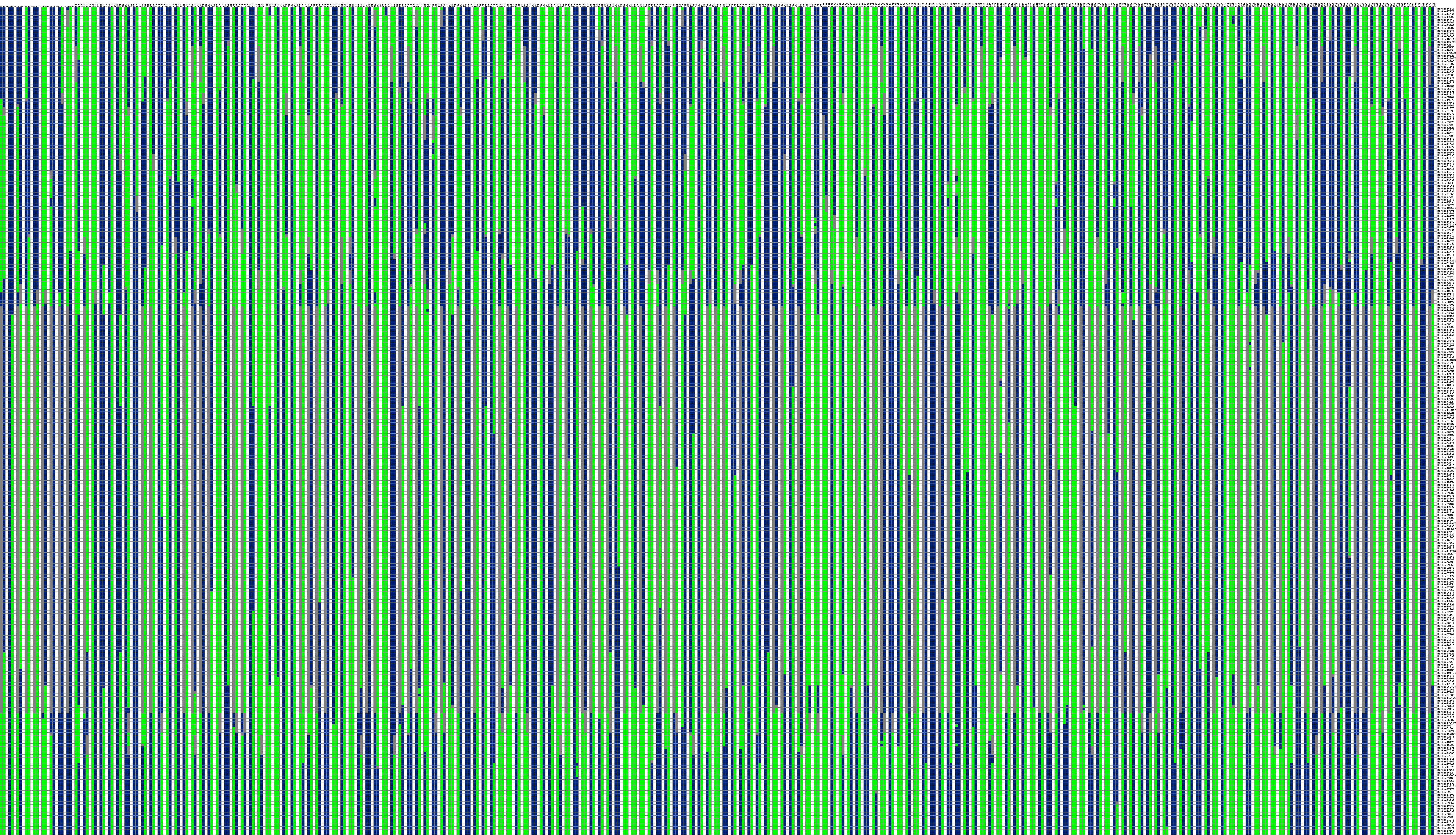

Supplement: Supplementary Material Presentation 1 — Haplotype map of the integrated maps. Each row represents a marker. Markers are ranked in accordance with the map order. Each of the two columns represents an individual; blank columns are used between two individuals. The first and second columns represent the paternal and maternal chromosomes, respectively. The green and blue areas in the columns represent the first and second alleles from the parents, respectively. The white column represents the source of alleles that cannot be judged. The gray areas represent the deleted alleles. [file Presentation1.ZIP › Supplementary Material Presentation 1/LG3.haploMap.png]

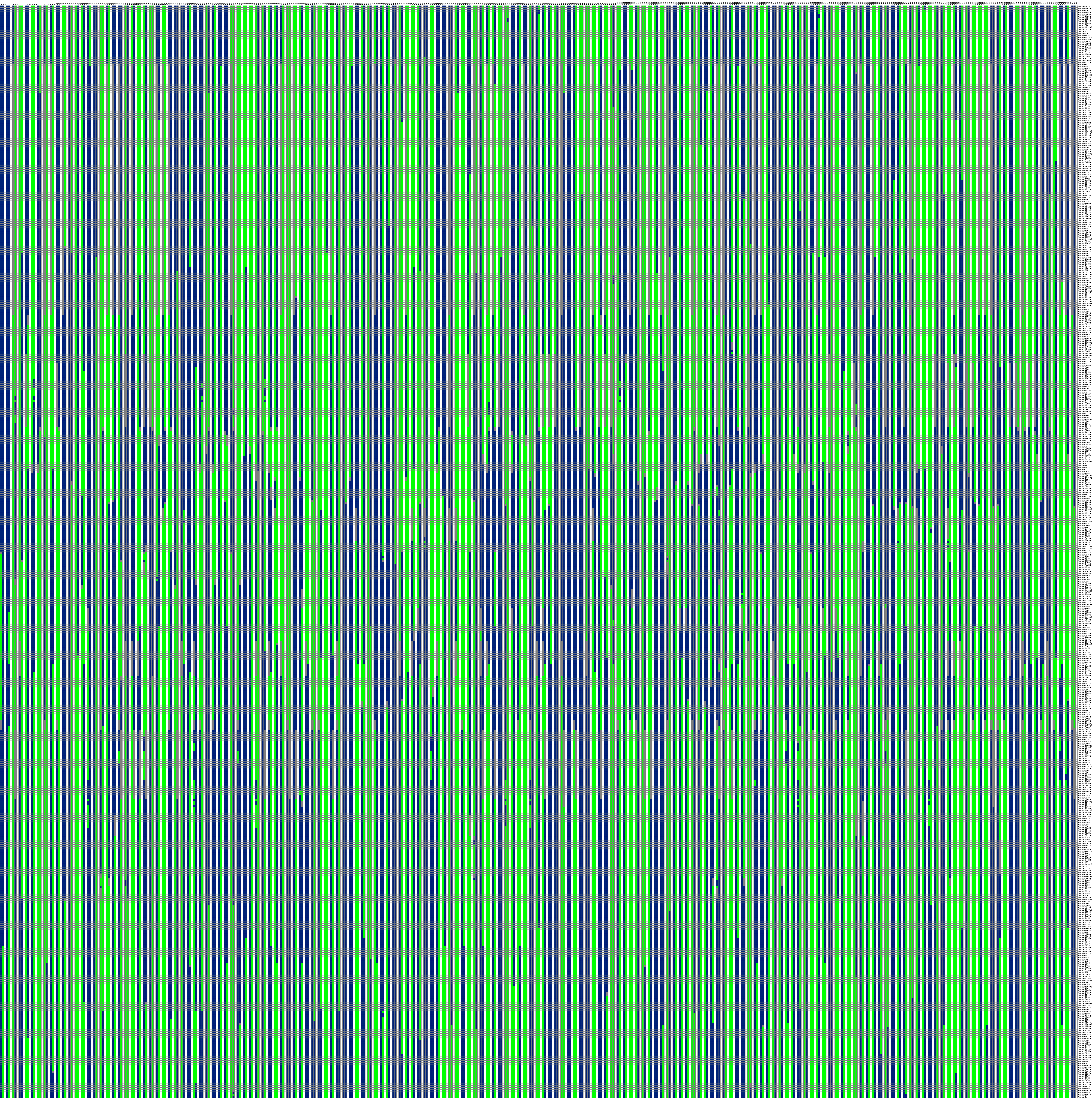

Supplement: Supplementary Material Presentation 1 — Haplotype map of the integrated maps. Each row represents a marker. Markers are ranked in accordance with the map order. Each of the two columns represents an individual; blank columns are used between two individuals. The first and second columns represent the paternal and maternal chromosomes, respectively. The green and blue areas in the columns represent the first and second alleles from the parents, respectively. The white column represents the source of alleles that cannot be judged. The gray areas represent the deleted alleles. [file Presentation1.ZIP › Supplementary Material Presentation 1/LG4.haploMap.png]

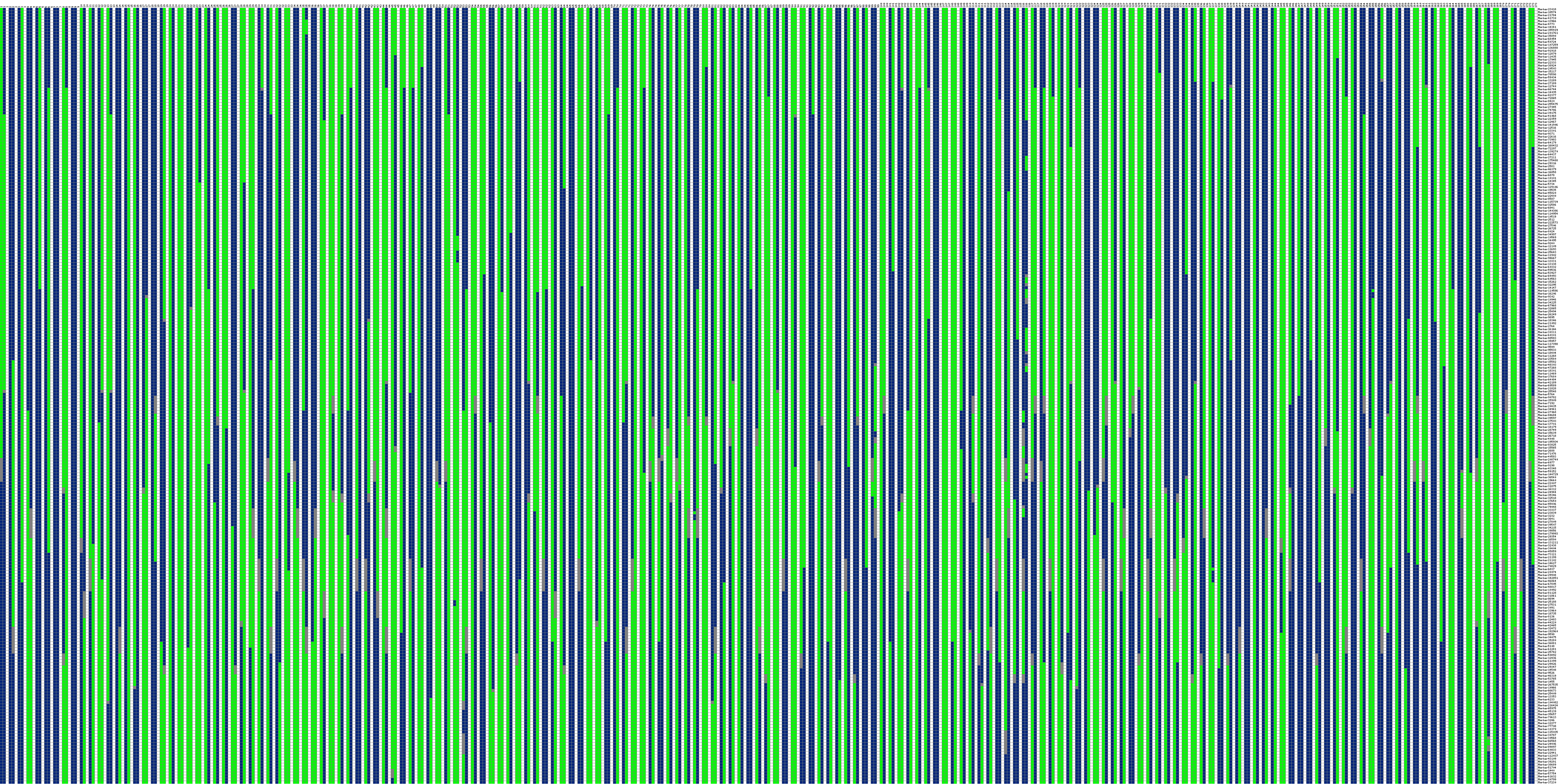

Supplement: Supplementary Material Presentation 1 — Haplotype map of the integrated maps. Each row represents a marker. Markers are ranked in accordance with the map order. Each of the two columns represents an individual; blank columns are used between two individuals. The first and second columns represent the paternal and maternal chromosomes, respectively. The green and blue areas in the columns represent the first and second alleles from the parents, respectively. The white column represents the source of alleles that cannot be judged. The gray areas represent the deleted alleles. [file Presentation1.ZIP › Supplementary Material Presentation 1/LG5.haploMap.png]

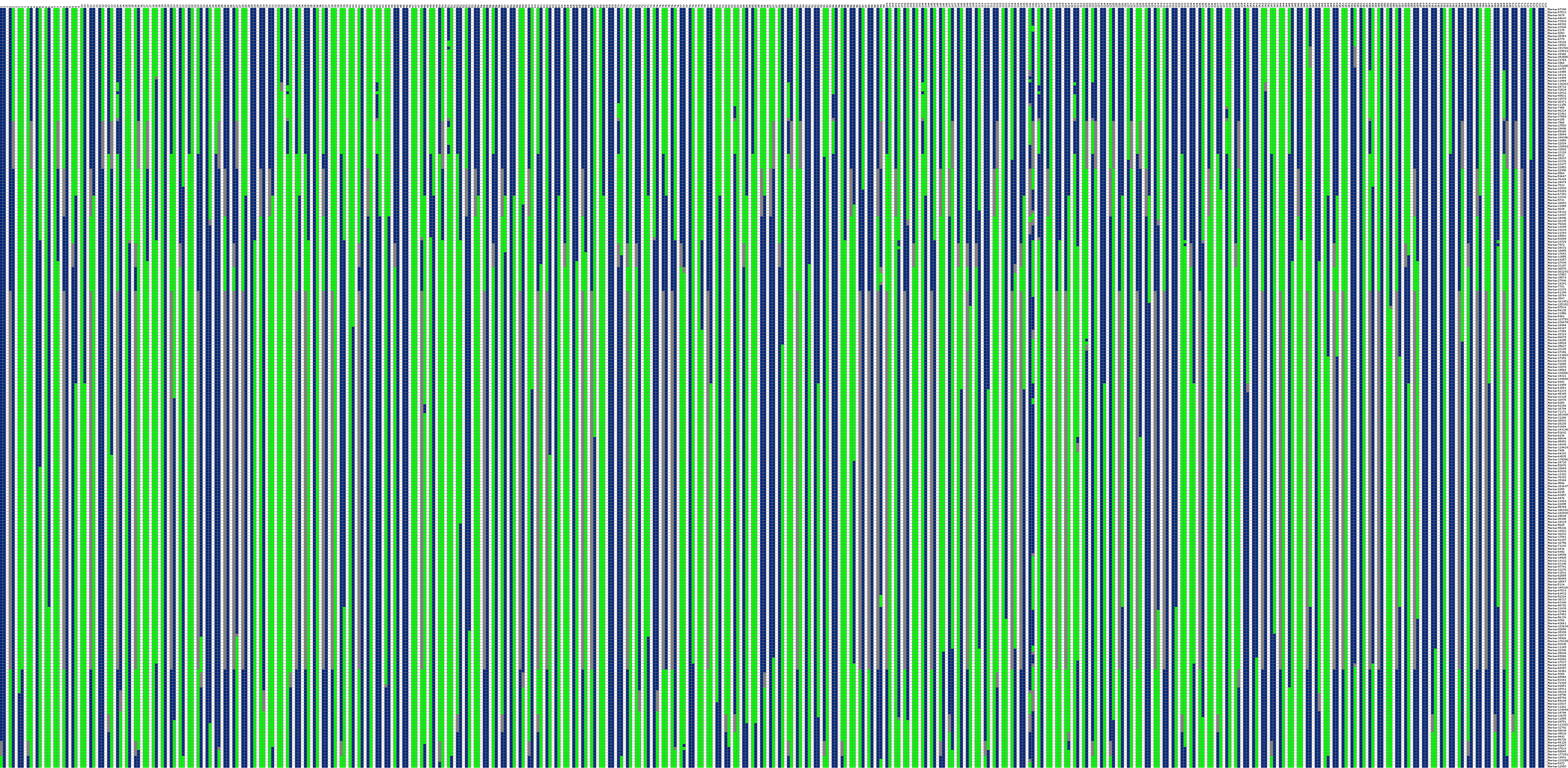

Supplement: Supplementary Material Presentation 1 — Haplotype map of the integrated maps. Each row represents a marker. Markers are ranked in accordance with the map order. Each of the two columns represents an individual; blank columns are used between two individuals. The first and second columns represent the paternal and maternal chromosomes, respectively. The green and blue areas in the columns represent the first and second alleles from the parents, respectively. The white column represents the source of alleles that cannot be judged. The gray areas represent the deleted alleles. [file Presentation1.ZIP › Supplementary Material Presentation 1/LG6.haploMap.png]

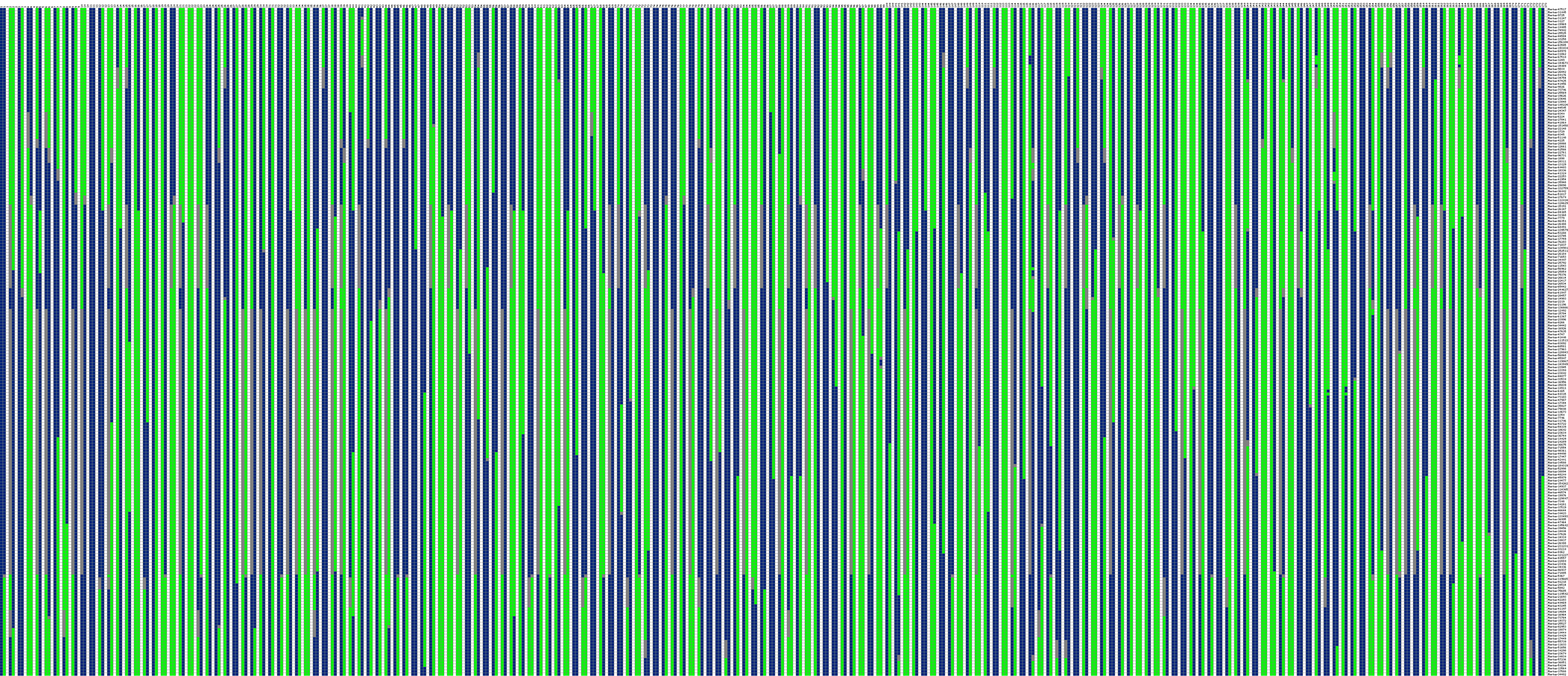

Supplement: Supplementary Material Presentation 1 — Haplotype map of the integrated maps. Each row represents a marker. Markers are ranked in accordance with the map order. Each of the two columns represents an individual; blank columns are used between two individuals. The first and second columns represent the paternal and maternal chromosomes, respectively. The green and blue areas in the columns represent the first and second alleles from the parents, respectively. The white column represents the source of alleles that cannot be judged. The gray areas represent the deleted alleles. [file Presentation1.ZIP › Supplementary Material Presentation 1/LG7.haploMap.png]

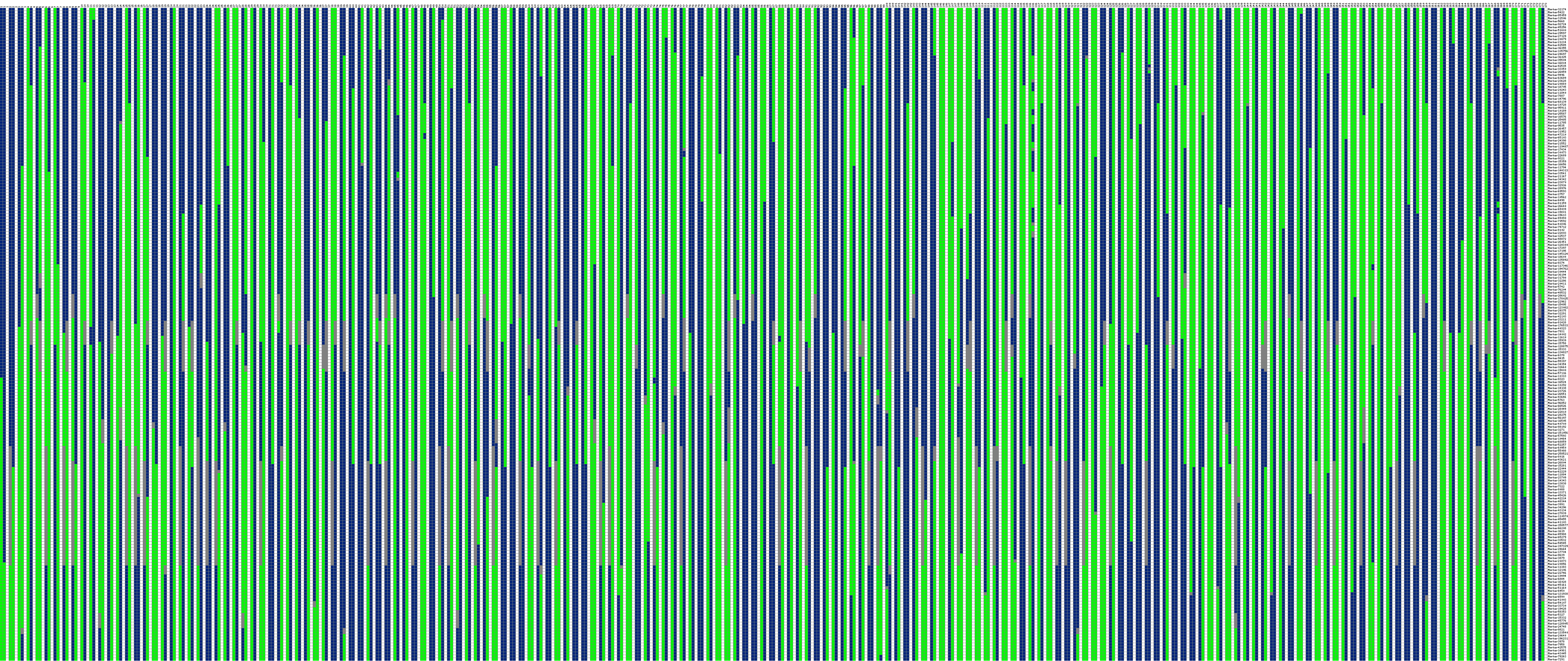

Supplement: Supplementary Material Presentation 1 — Haplotype map of the integrated maps. Each row represents a marker. Markers are ranked in accordance with the map order. Each of the two columns represents an individual; blank columns are used between two individuals. The first and second columns represent the paternal and maternal chromosomes, respectively. The green and blue areas in the columns represent the first and second alleles from the parents, respectively. The white column represents the source of alleles that cannot be judged. The gray areas represent the deleted alleles. [file Presentation1.ZIP › Supplementary Material Presentation 1/LG8.haploMap.png]

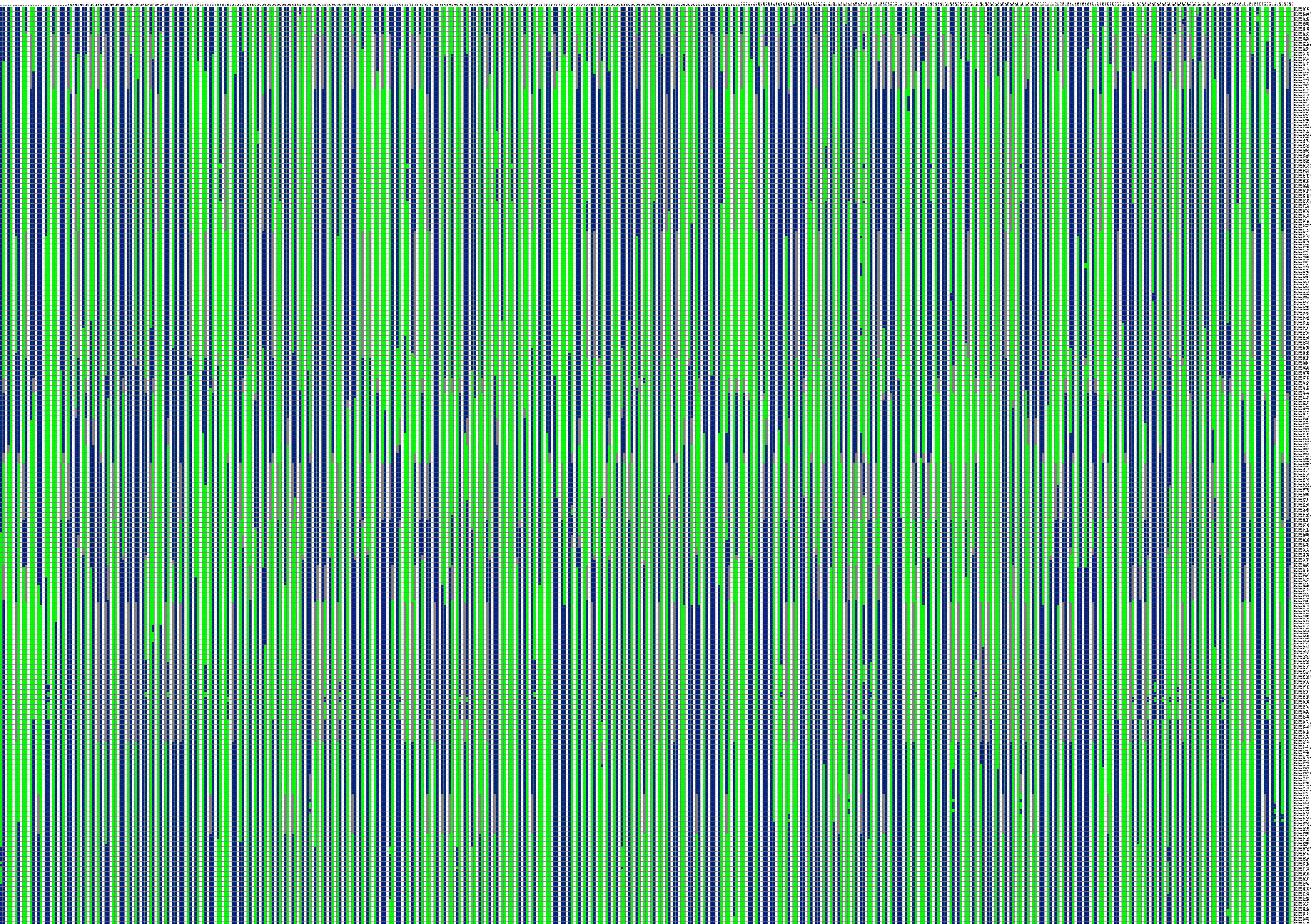

Supplement: Supplementary Material Presentation 1 — Haplotype map of the integrated maps. Each row represents a marker. Markers are ranked in accordance with the map order. Each of the two columns represents an individual; blank columns are used between two individuals. The first and second columns represent the paternal and maternal chromosomes, respectively. The green and blue areas in the columns represent the first and second alleles from the parents, respectively. The white column represents the source of alleles that cannot be judged. The gray areas represent the deleted alleles. [file Presentation1.ZIP › Supplementary Material Presentation 1/LG9.haploMap.png]

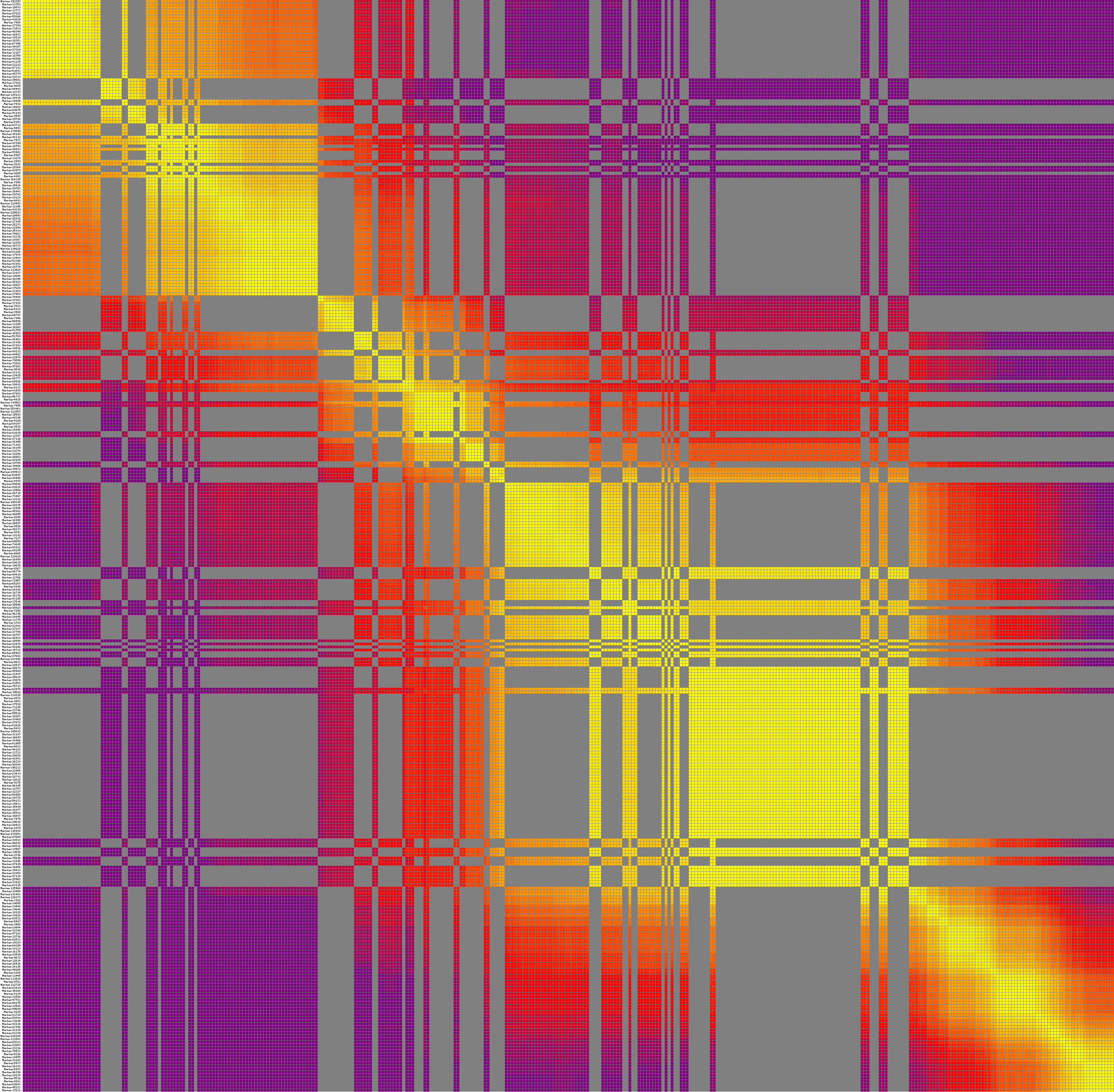

Supplement: Supplementary Material Presentation 2 — Heat map of the integrated maps. Markers of each row and column are ranked according to the map order; each small square represents the rate of recombination (r) between the two markers. [file Presentation2.ZIP › Supplementary Material Presentation 2/LG1.heatMap.png]

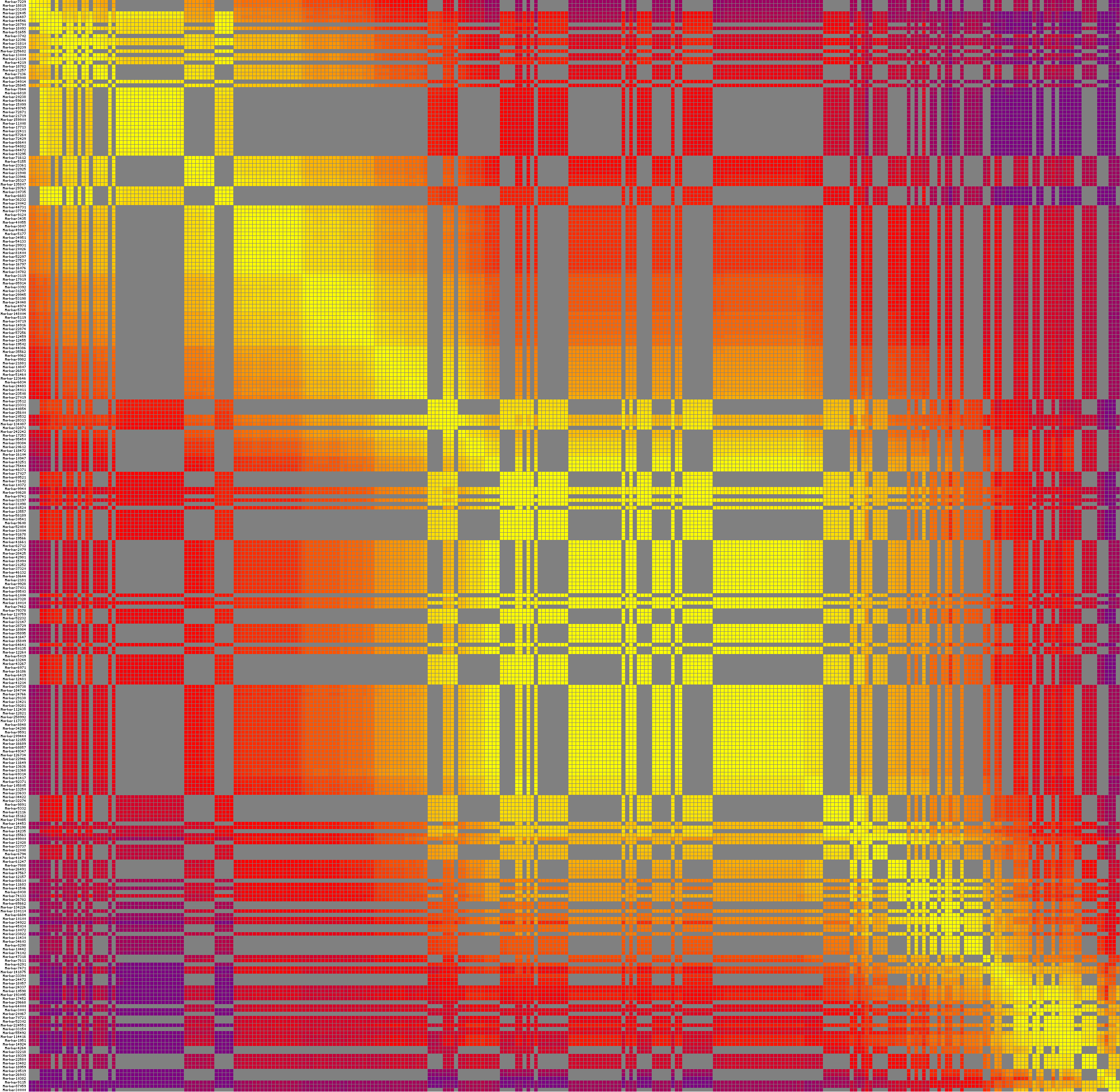

Supplement: Supplementary Material Presentation 2 — Heat map of the integrated maps. Markers of each row and column are ranked according to the map order; each small square represents the rate of recombination (r) between the two markers. [file Presentation2.ZIP › Supplementary Material Presentation 2/LG10.heatMap.png]

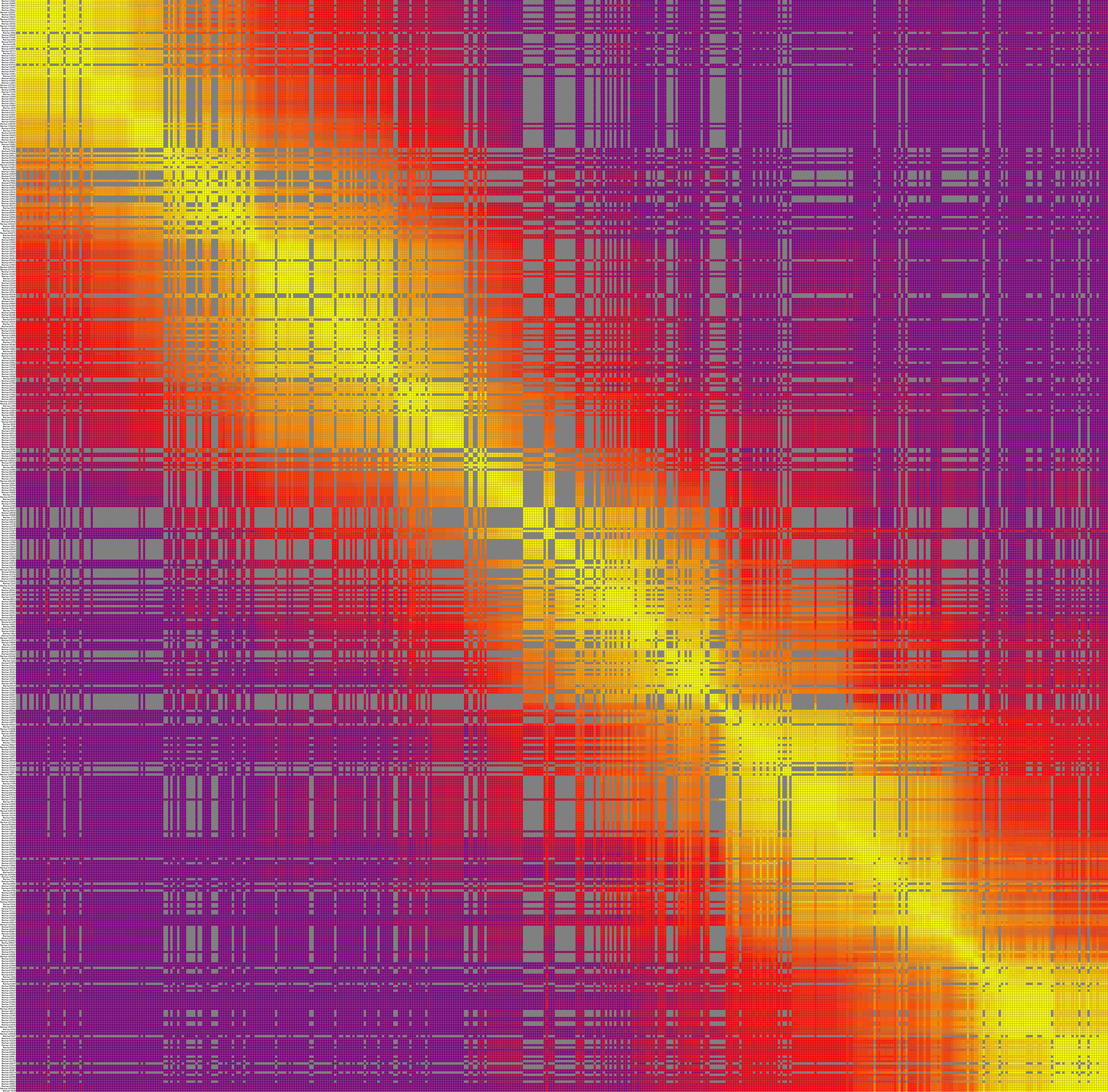

Supplement: Supplementary Material Presentation 2 — Heat map of the integrated maps. Markers of each row and column are ranked according to the map order; each small square represents the rate of recombination (r) between the two markers. [file Presentation2.ZIP › Supplementary Material Presentation 2/LG11.heatMap.png]

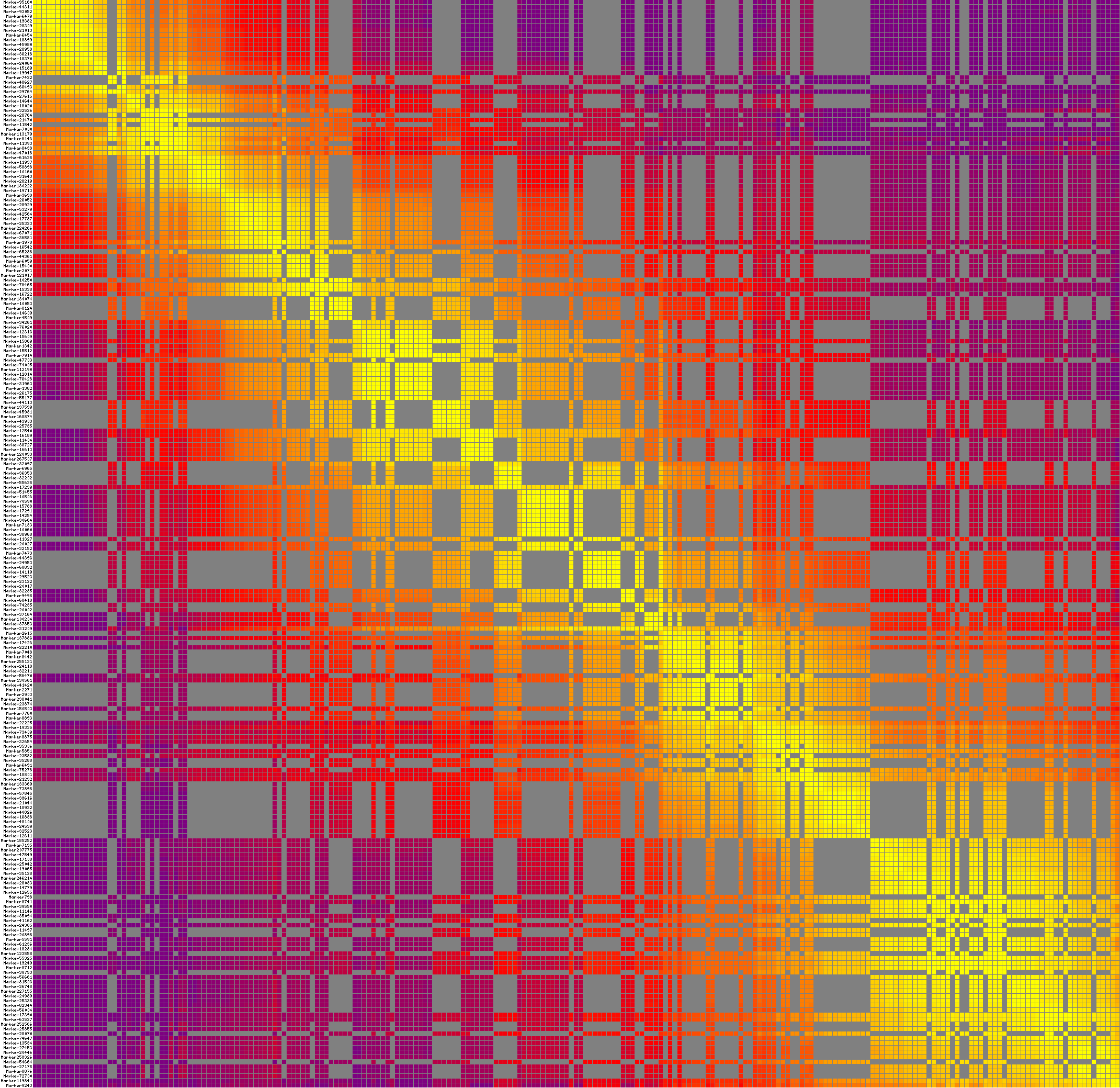

Supplement: Supplementary Material Presentation 2 — Heat map of the integrated maps. Markers of each row and column are ranked according to the map order; each small square represents the rate of recombination (r) between the two markers. [file Presentation2.ZIP › Supplementary Material Presentation 2/LG12.heatMap.png]

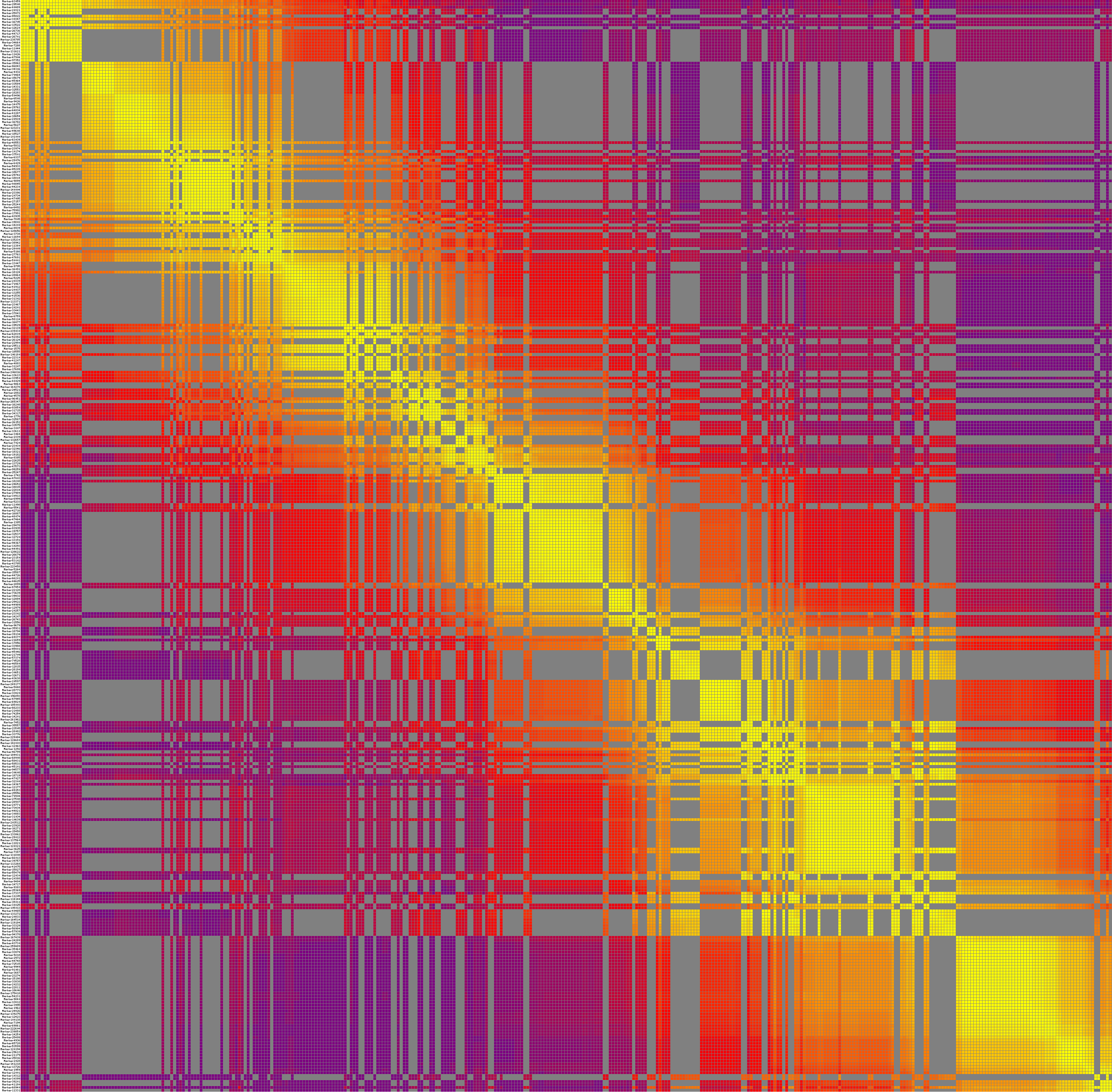

Supplement: Supplementary Material Presentation 2 — Heat map of the integrated maps. Markers of each row and column are ranked according to the map order; each small square represents the rate of recombination (r) between the two markers. [file Presentation2.ZIP › Supplementary Material Presentation 2/LG13.heatMap.png]

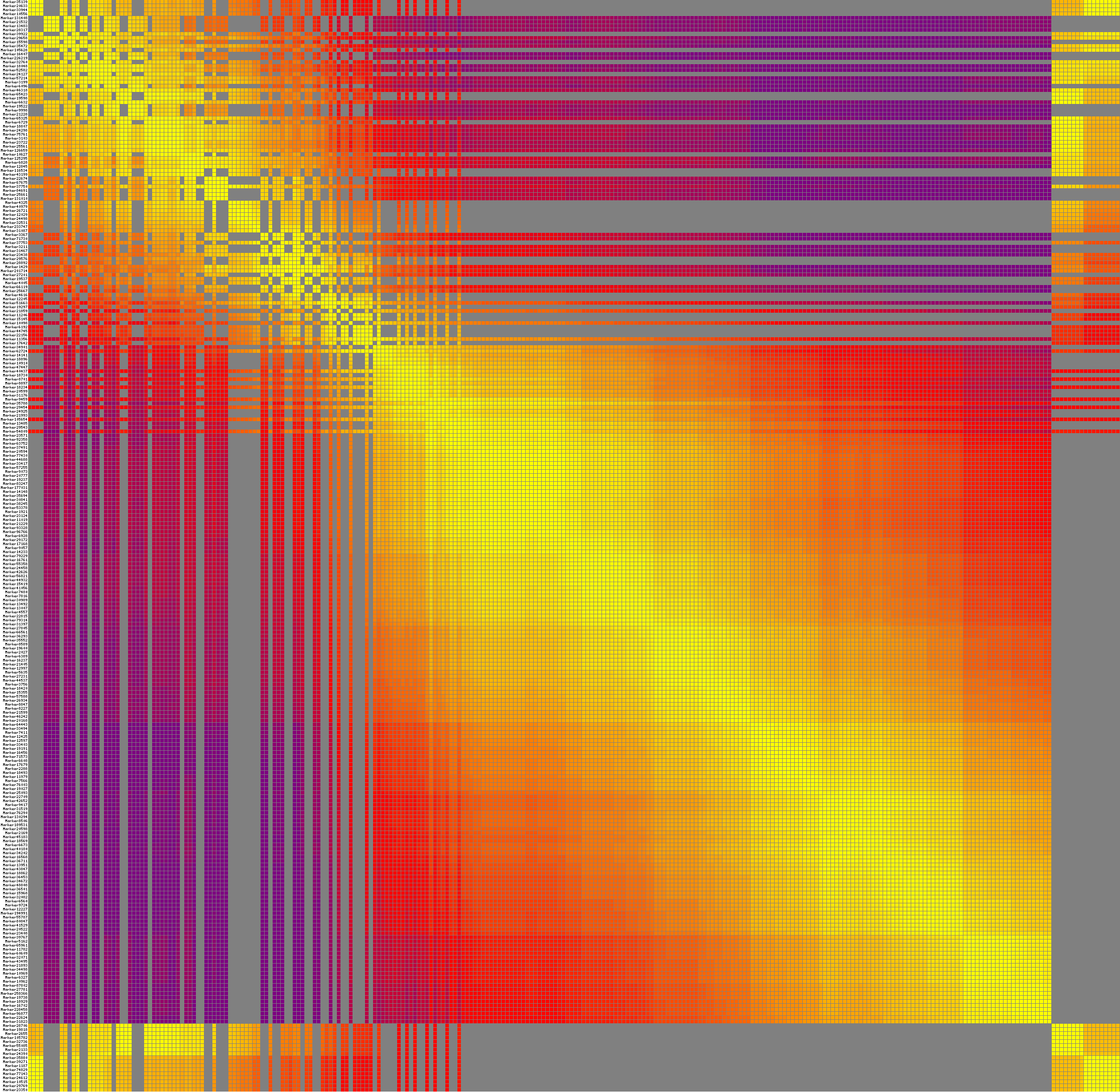

Supplement: Supplementary Material Presentation 2 — Heat map of the integrated maps. Markers of each row and column are ranked according to the map order; each small square represents the rate of recombination (r) between the two markers. [file Presentation2.ZIP › Supplementary Material Presentation 2/LG14.heatMap.png]

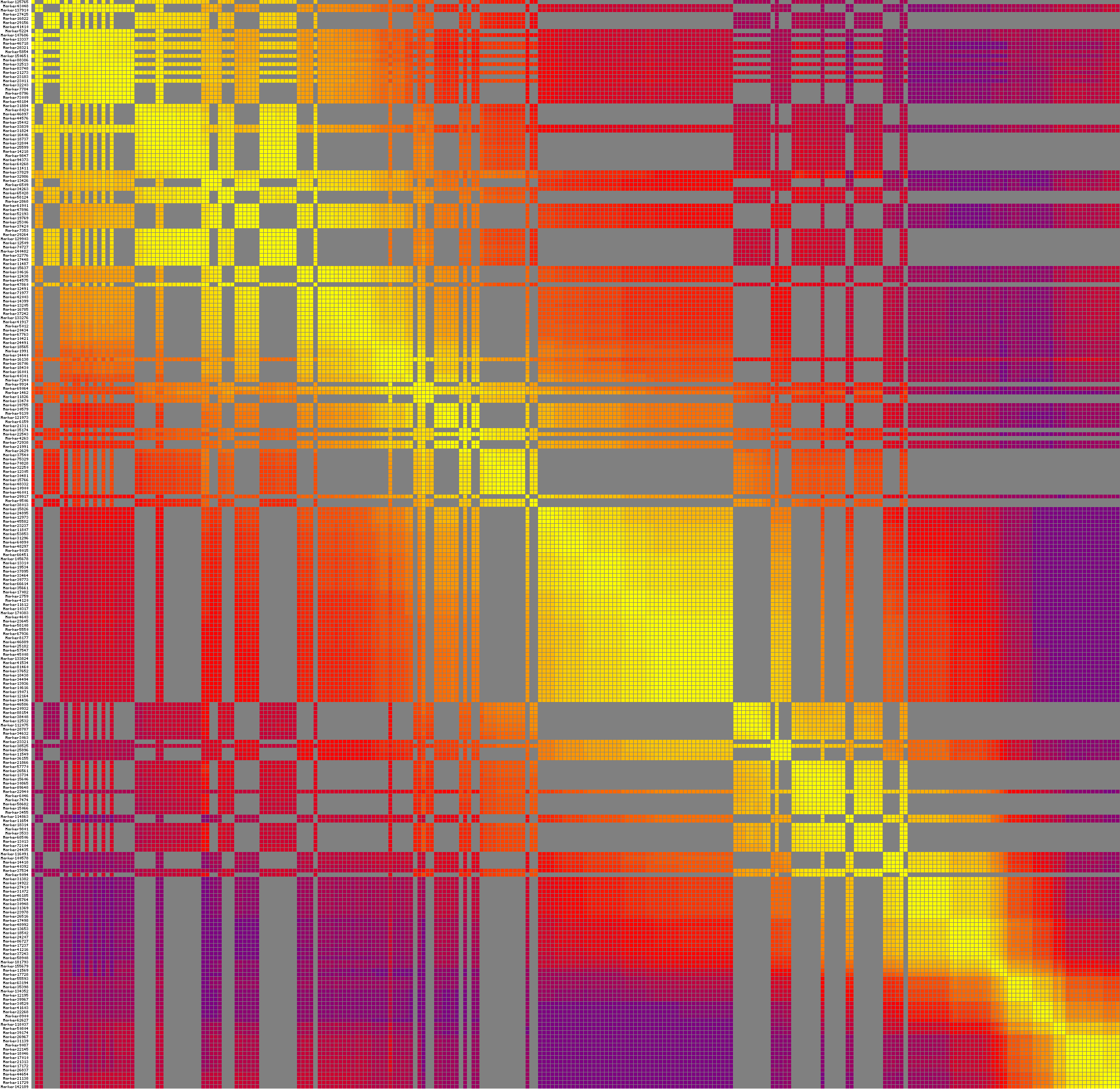

Supplement: Supplementary Material Presentation 2 — Heat map of the integrated maps. Markers of each row and column are ranked according to the map order; each small square represents the rate of recombination (r) between the two markers. [file Presentation2.ZIP › Supplementary Material Presentation 2/LG15.heatMap.png]

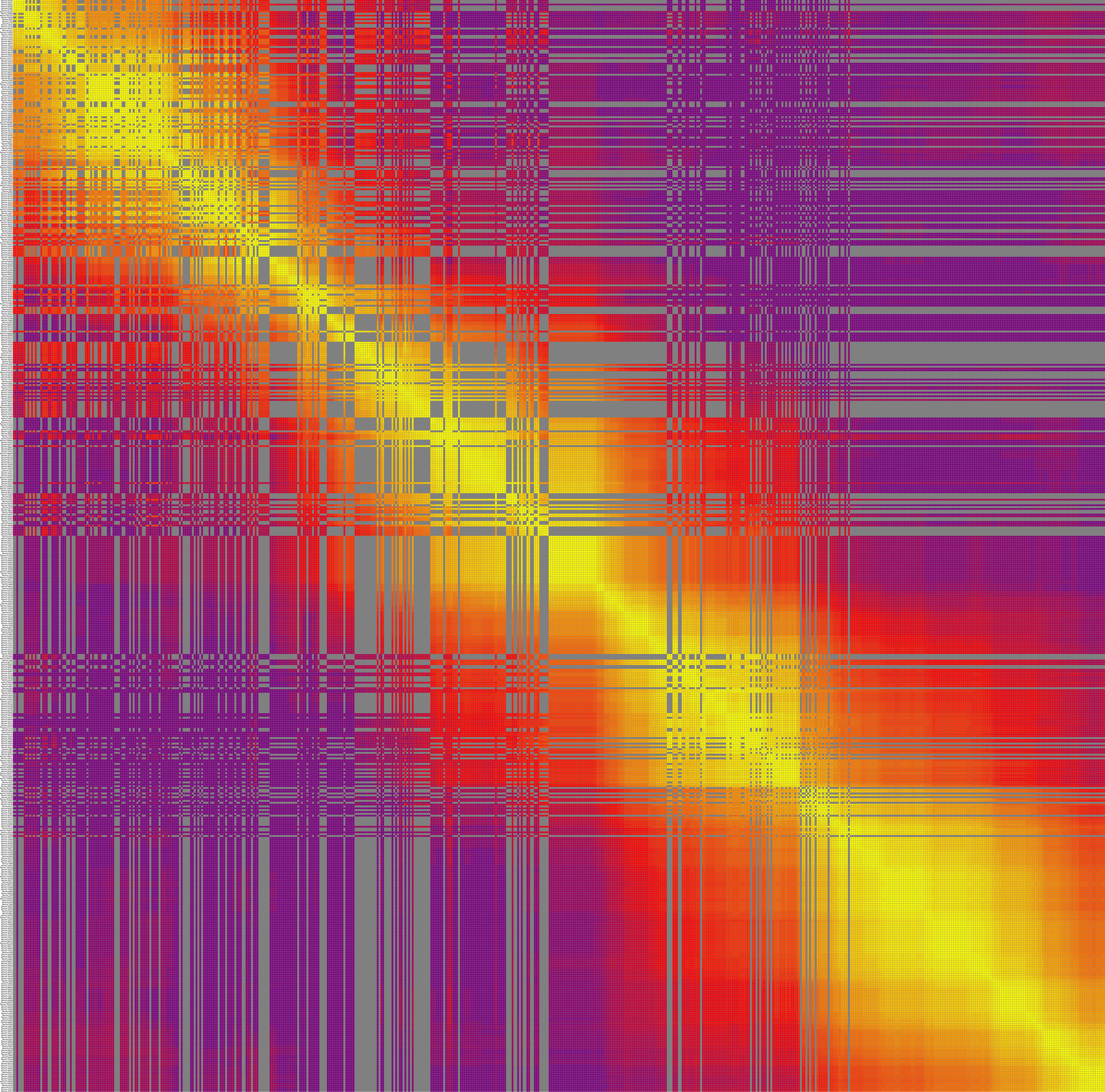

Supplement: Supplementary Material Presentation 2 — Heat map of the integrated maps. Markers of each row and column are ranked according to the map order; each small square represents the rate of recombination (r) between the two markers. [file Presentation2.ZIP › Supplementary Material Presentation 2/LG16.heatMap.png]

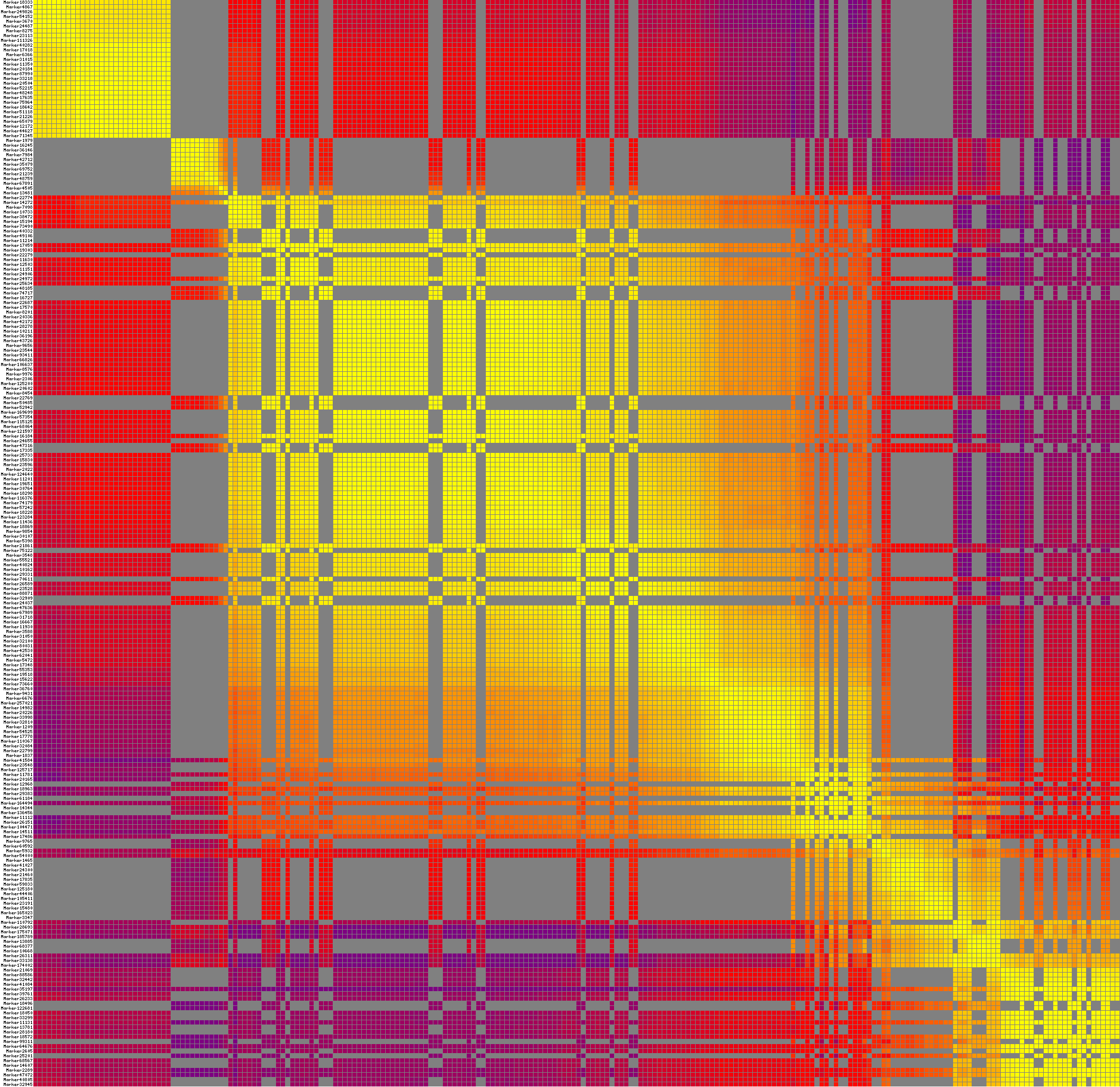

Supplement: Supplementary Material Presentation 2 — Heat map of the integrated maps. Markers of each row and column are ranked according to the map order; each small square represents the rate of recombination (r) between the two markers. [file Presentation2.ZIP › Supplementary Material Presentation 2/LG17.heatMap.png]

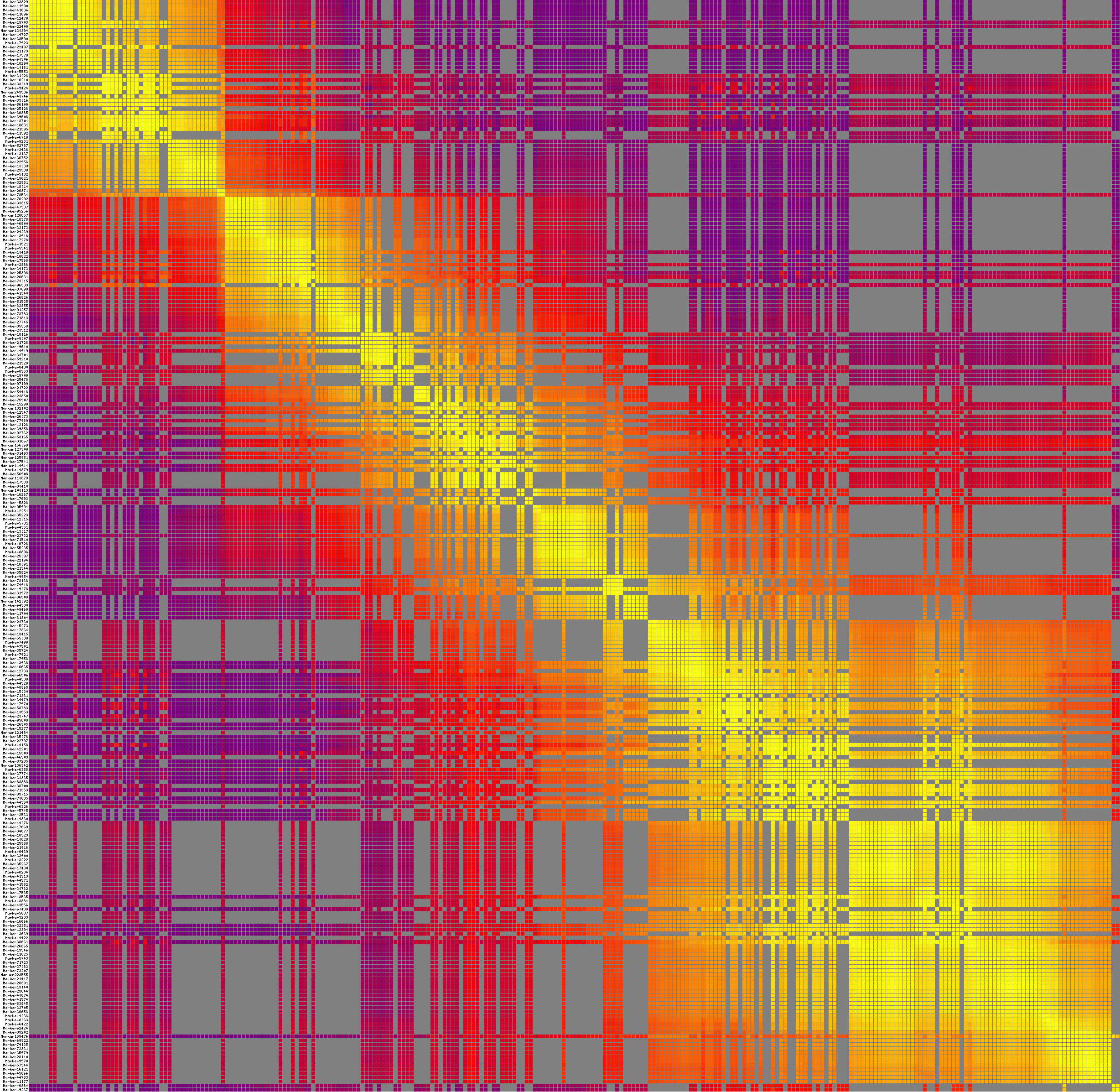

Supplement: Supplementary Material Presentation 2 — Heat map of the integrated maps. Markers of each row and column are ranked according to the map order; each small square represents the rate of recombination (r) between the two markers. [file Presentation2.ZIP › Supplementary Material Presentation 2/LG18.heatMap.png]

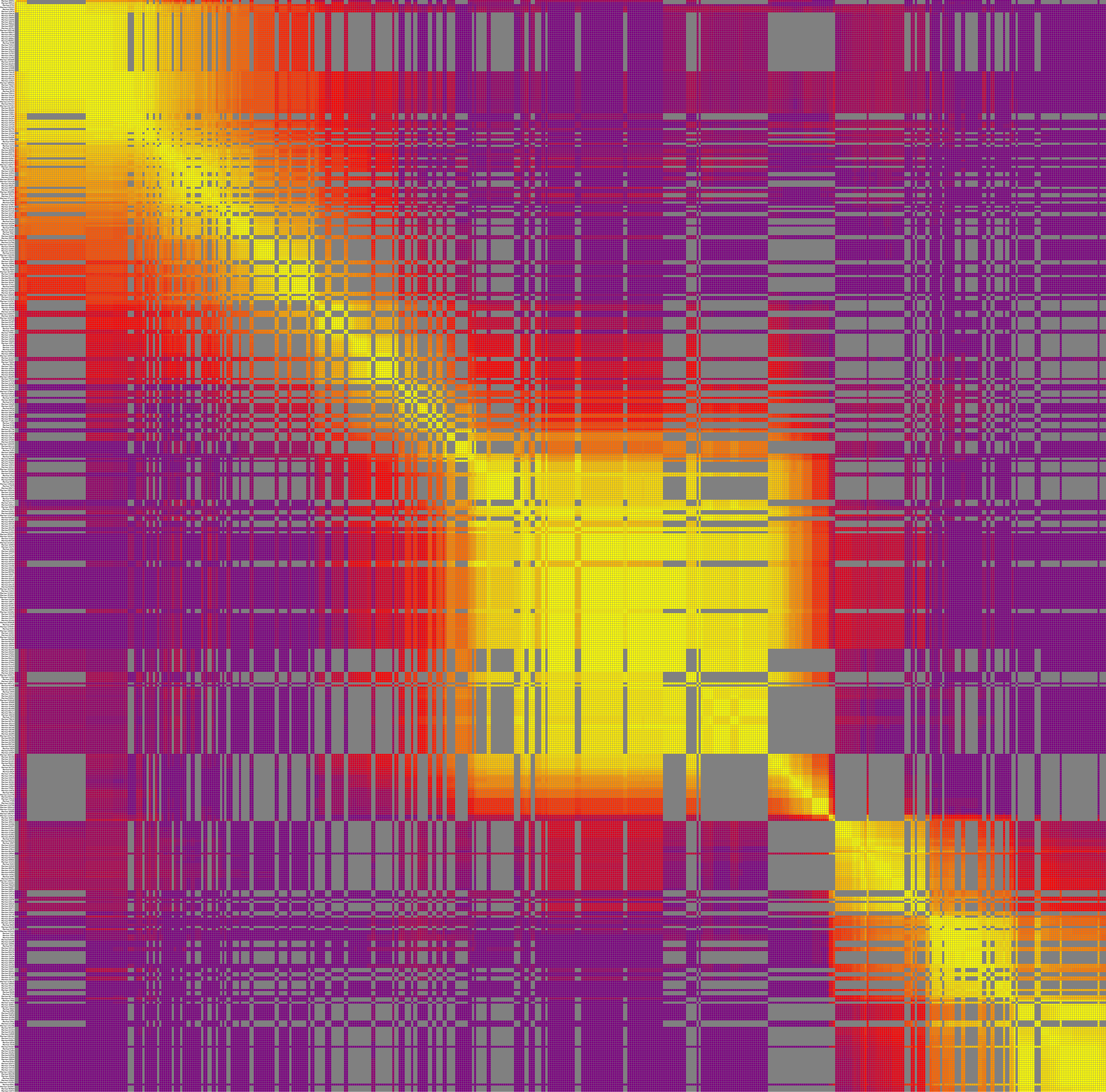

Supplement: Supplementary Material Presentation 2 — Heat map of the integrated maps. Markers of each row and column are ranked according to the map order; each small square represents the rate of recombination (r) between the two markers. [file Presentation2.ZIP › Supplementary Material Presentation 2/LG19.heatMap.png]

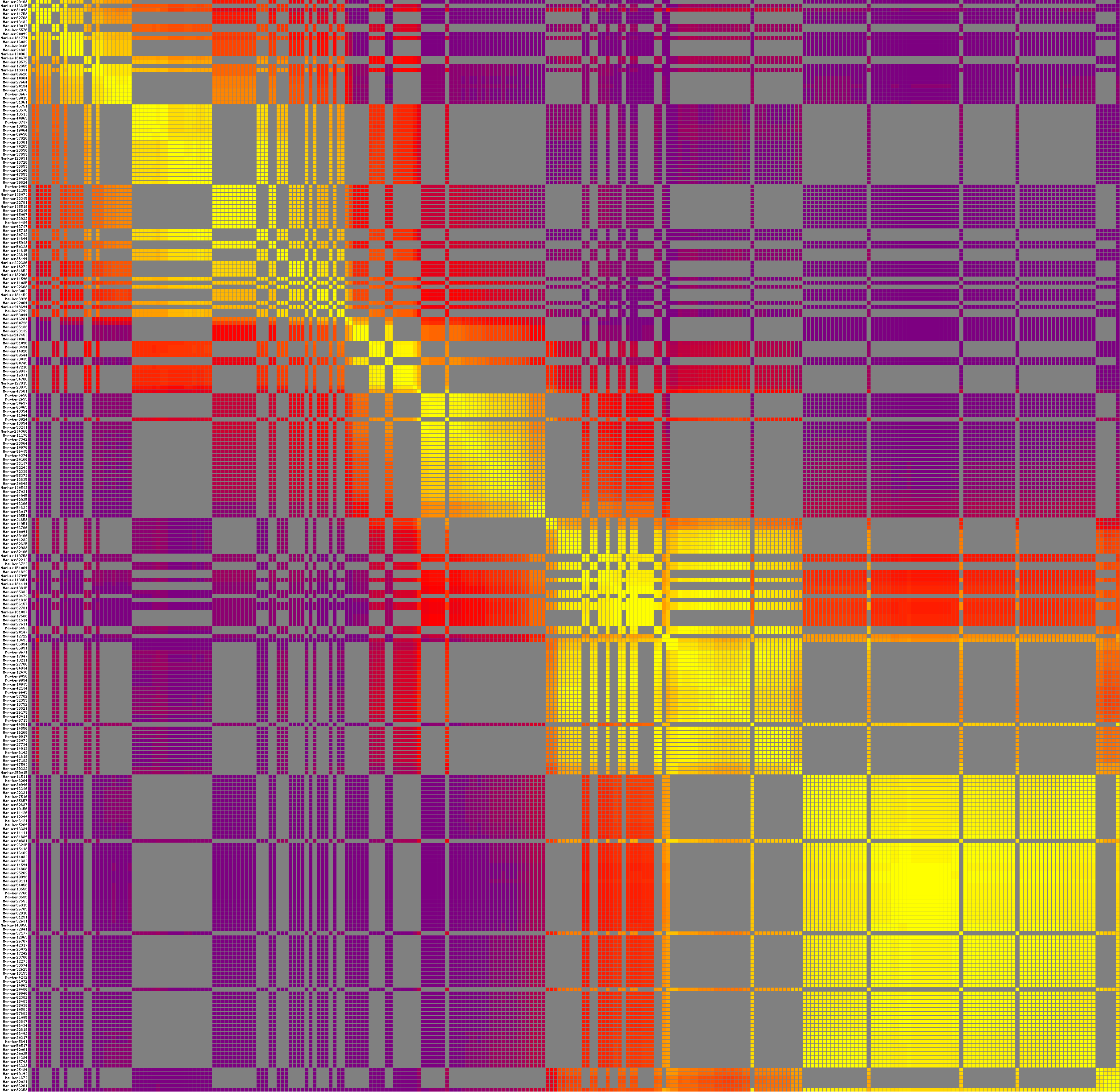

Supplement: Supplementary Material Presentation 2 — Heat map of the integrated maps. Markers of each row and column are ranked according to the map order; each small square represents the rate of recombination (r) between the two markers. [file Presentation2.ZIP › Supplementary Material Presentation 2/LG2.heatMap.png]

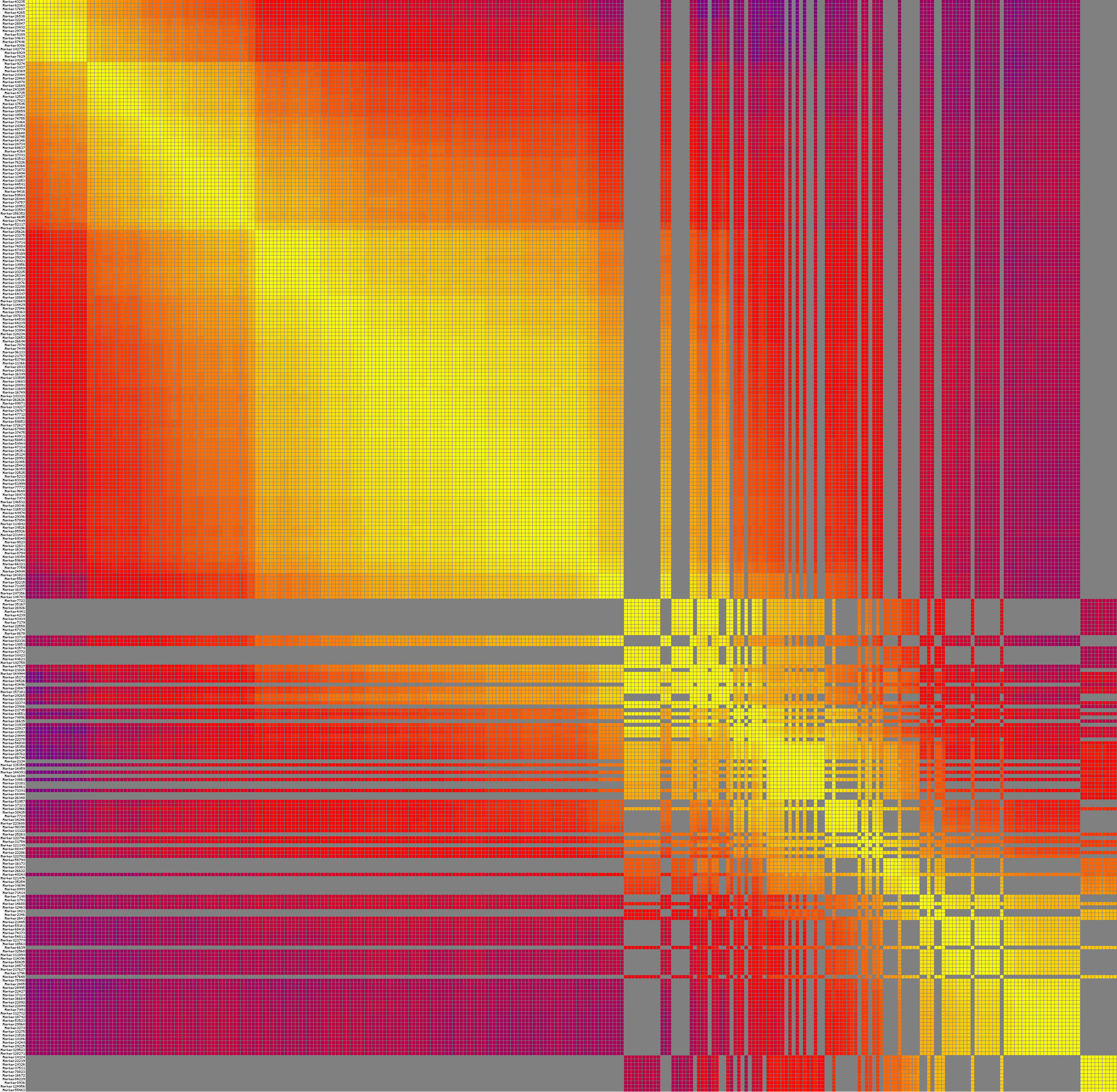

Supplement: Supplementary Material Presentation 2 — Heat map of the integrated maps. Markers of each row and column are ranked according to the map order; each small square represents the rate of recombination (r) between the two markers. [file Presentation2.ZIP › Supplementary Material Presentation 2/LG20.heatMap.png]

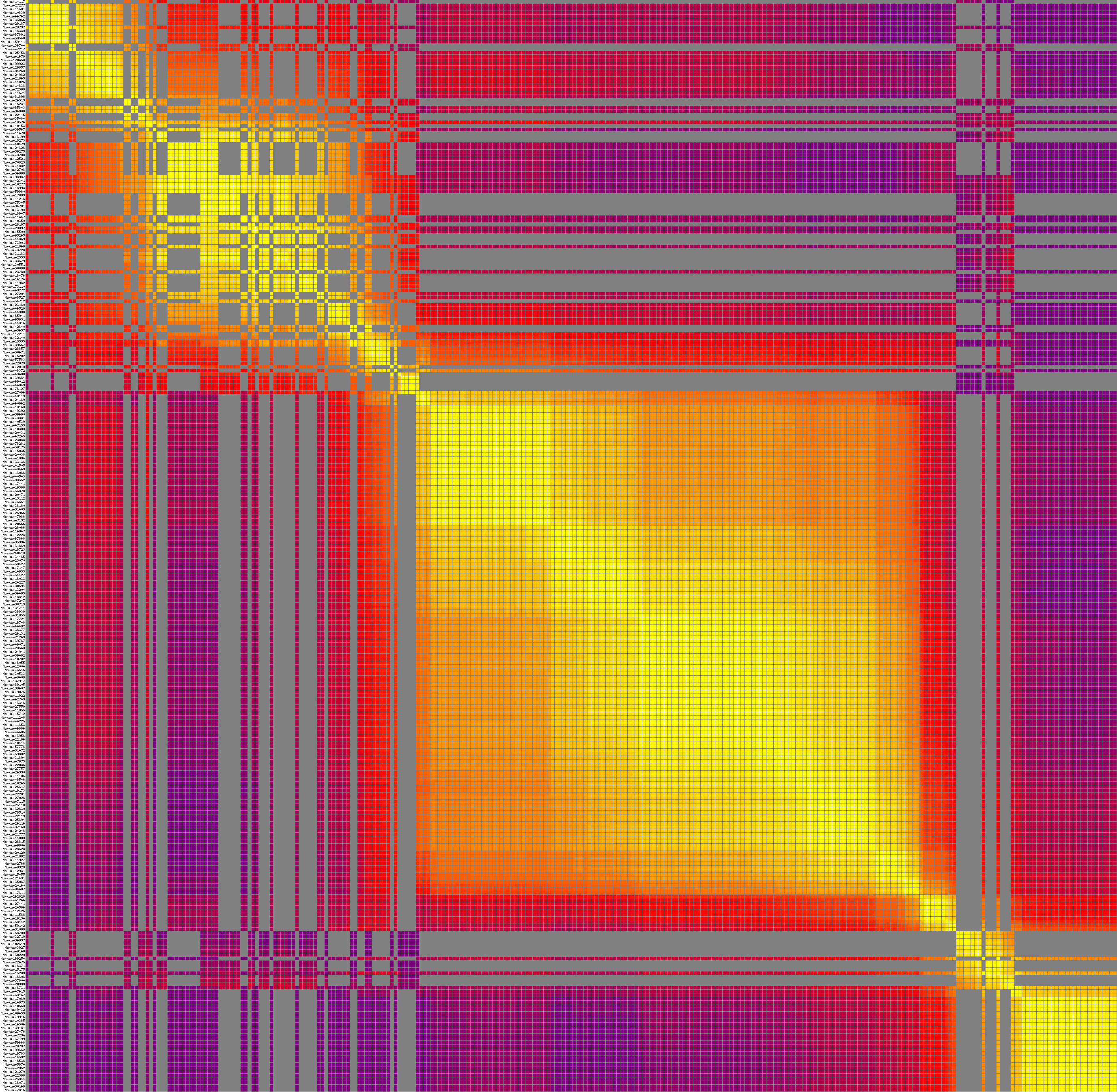

Supplement: Supplementary Material Presentation 2 — Heat map of the integrated maps. Markers of each row and column are ranked according to the map order; each small square represents the rate of recombination (r) between the two markers. [file Presentation2.ZIP › Supplementary Material Presentation 2/LG3.heatMap.png]

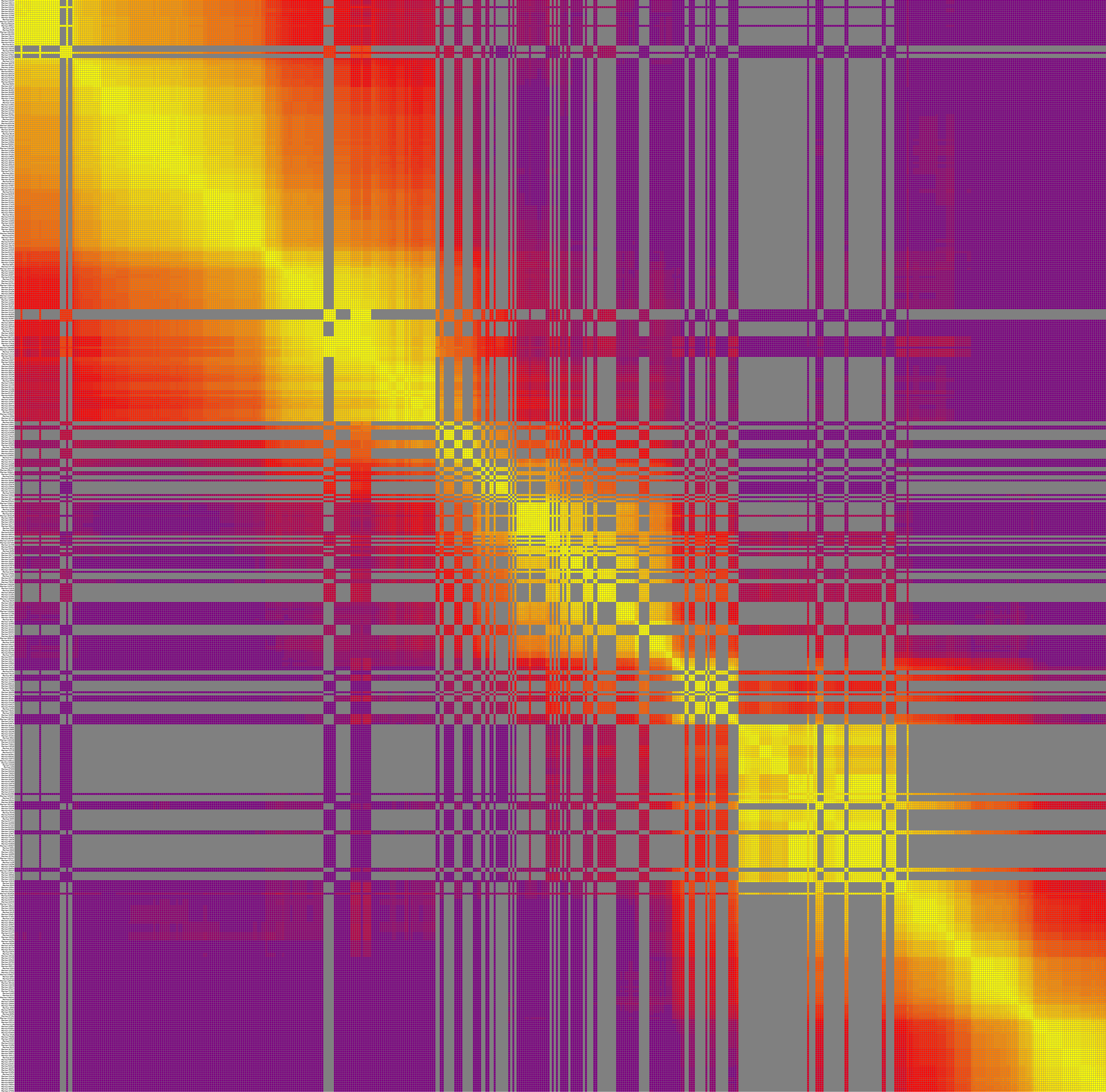

Supplement: Supplementary Material Presentation 2 — Heat map of the integrated maps. Markers of each row and column are ranked according to the map order; each small square represents the rate of recombination (r) between the two markers. [file Presentation2.ZIP › Supplementary Material Presentation 2/LG4.heatMap.png]

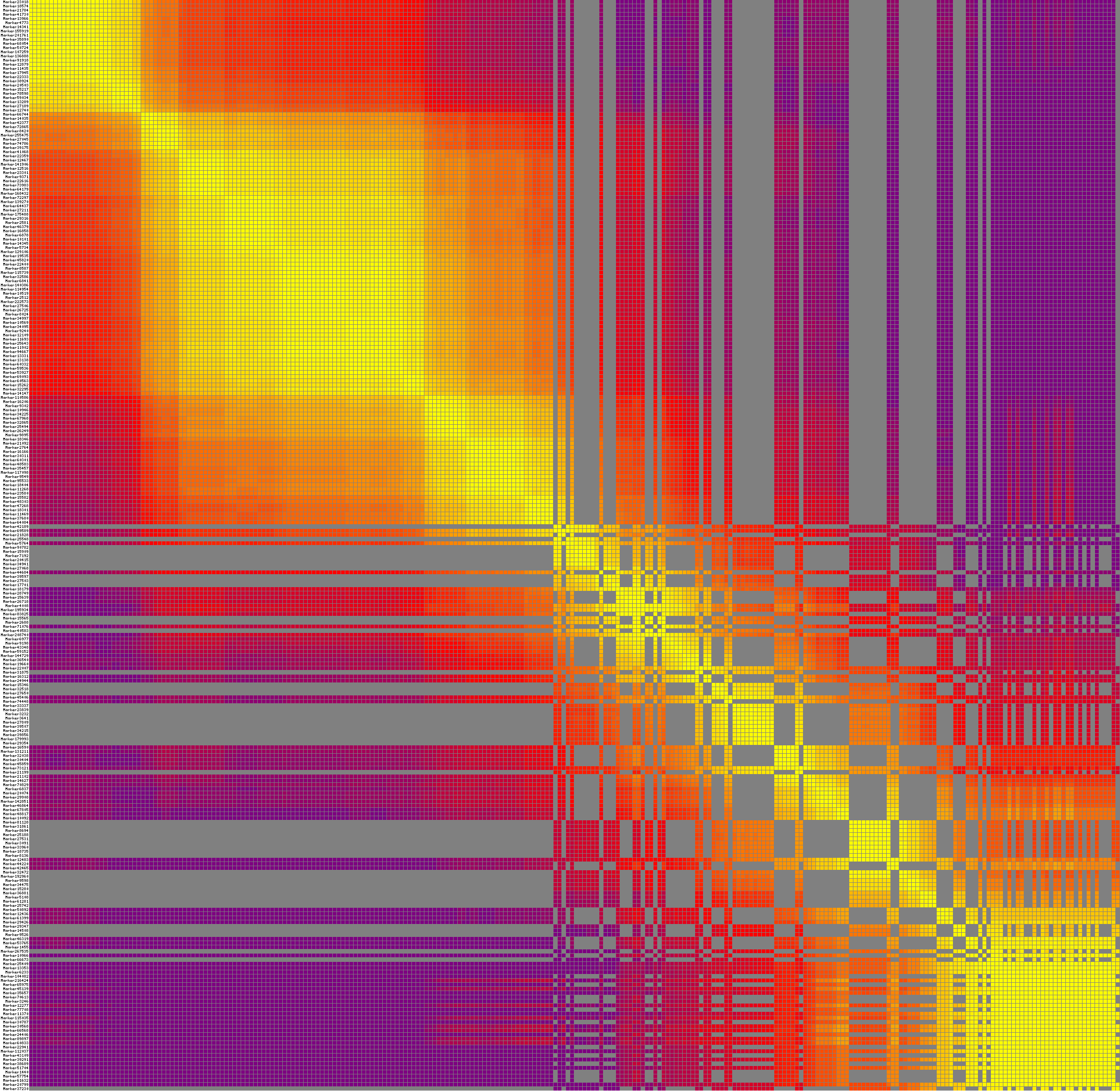

Supplement: Supplementary Material Presentation 2 — Heat map of the integrated maps. Markers of each row and column are ranked according to the map order; each small square represents the rate of recombination (r) between the two markers. [file Presentation2.ZIP › Supplementary Material Presentation 2/LG5.heatMap.png]

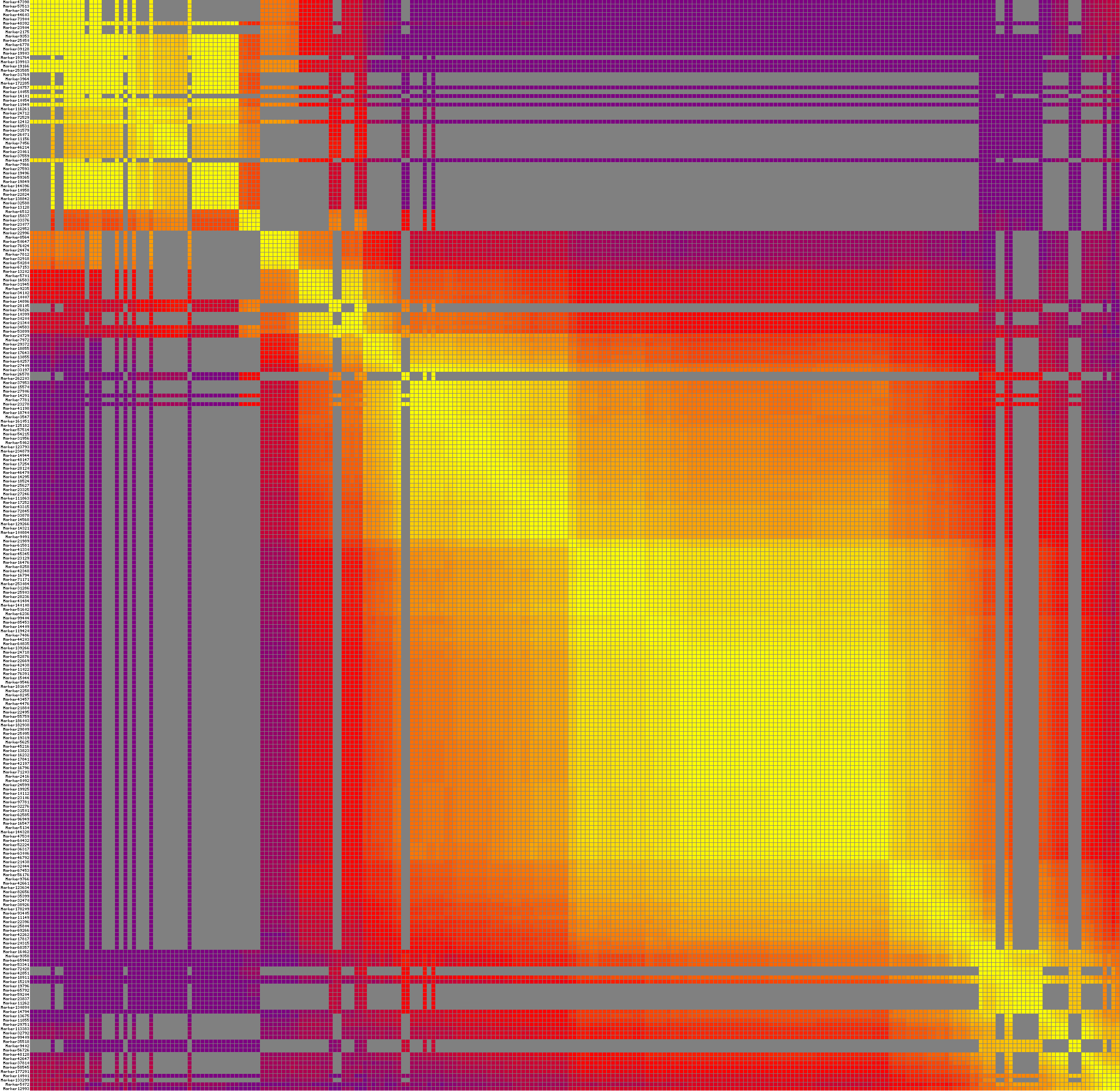

Supplement: Supplementary Material Presentation 2 — Heat map of the integrated maps. Markers of each row and column are ranked according to the map order; each small square represents the rate of recombination (r) between the two markers. [file Presentation2.ZIP › Supplementary Material Presentation 2/LG6.heatMap.png]

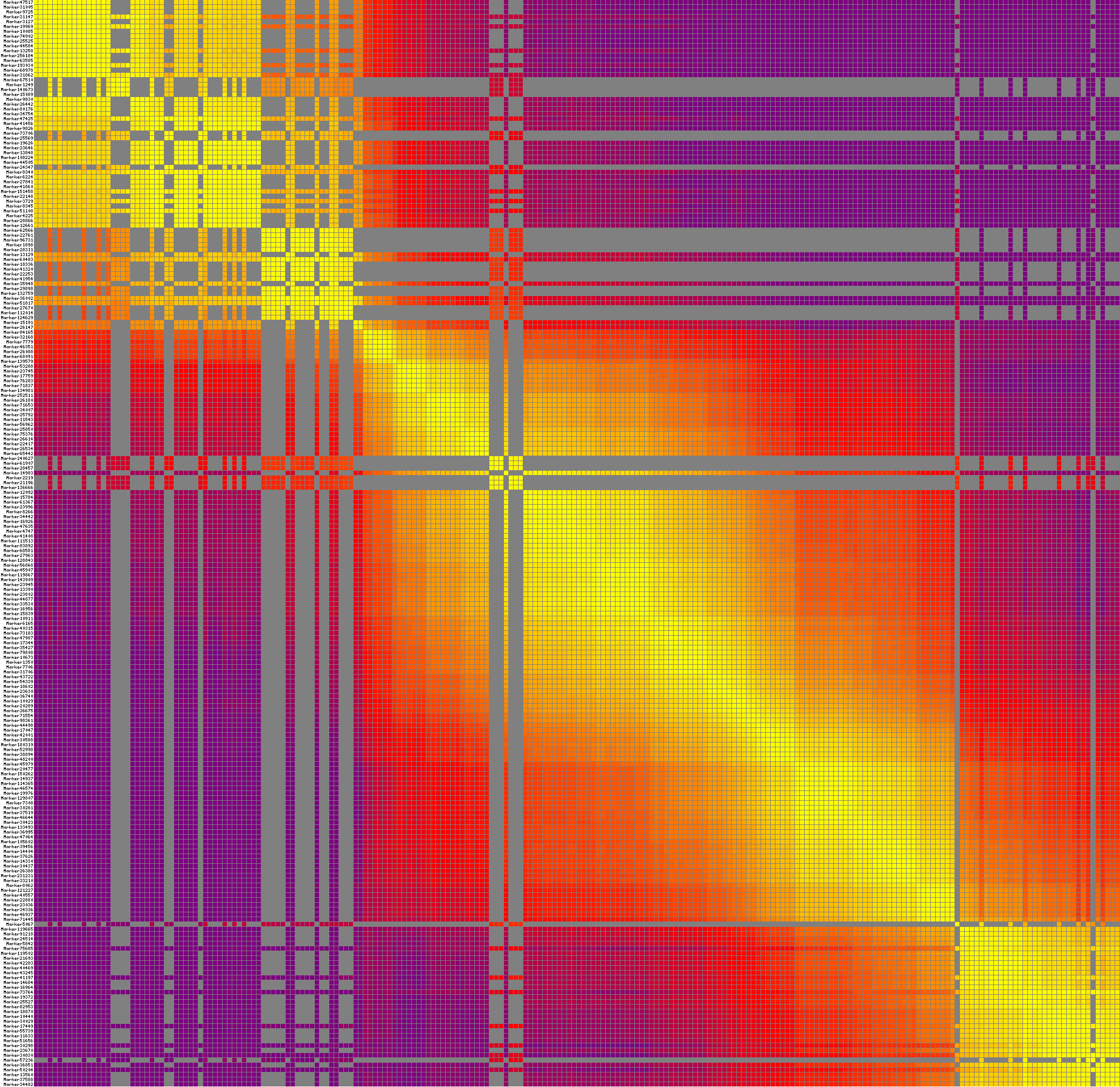

Supplement: Supplementary Material Presentation 2 — Heat map of the integrated maps. Markers of each row and column are ranked according to the map order; each small square represents the rate of recombination (r) between the two markers. [file Presentation2.ZIP › Supplementary Material Presentation 2/LG7.heatMap.png]

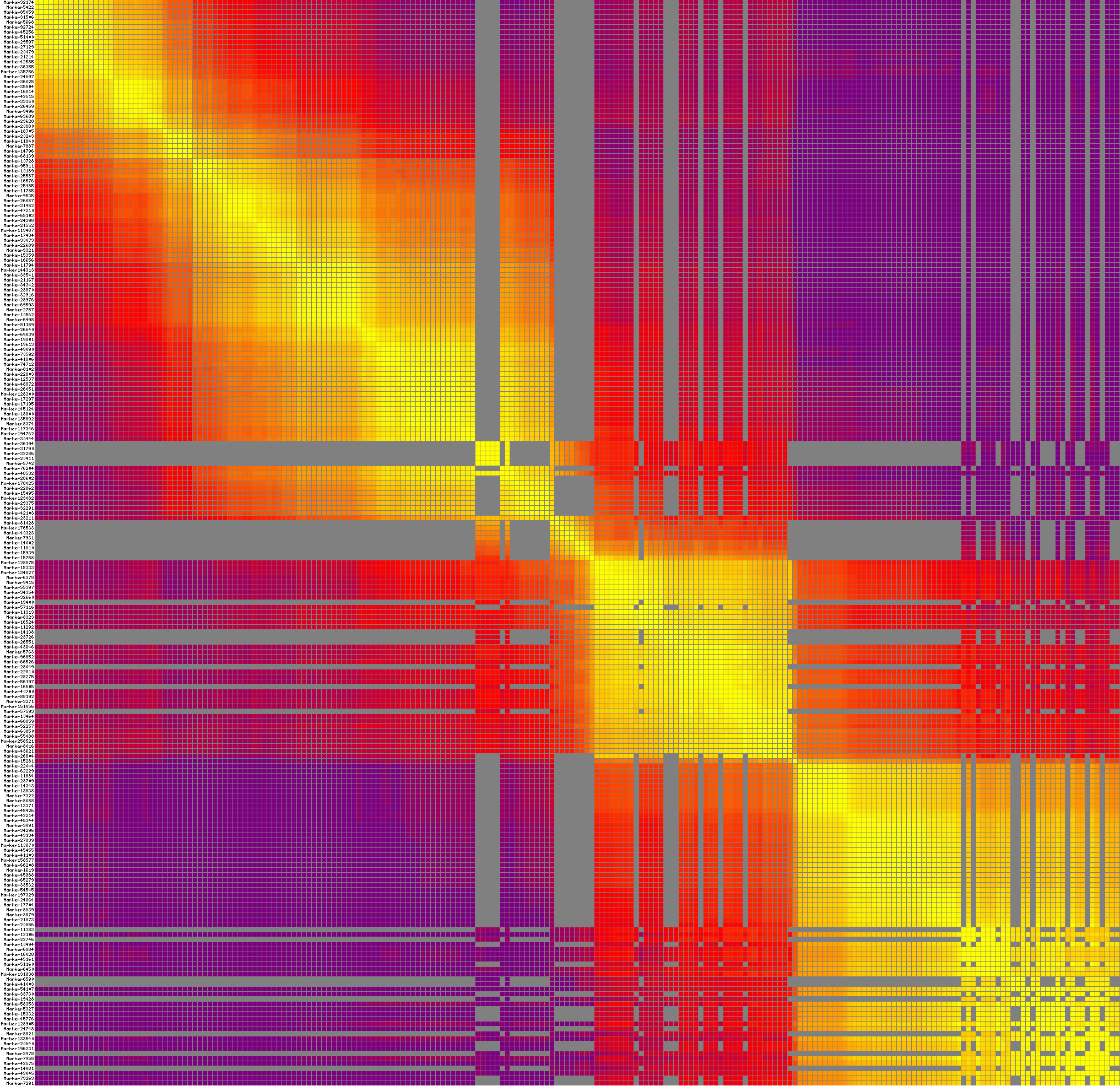

Supplement: Supplementary Material Presentation 2 — Heat map of the integrated maps. Markers of each row and column are ranked according to the map order; each small square represents the rate of recombination (r) between the two markers. [file Presentation2.ZIP › Supplementary Material Presentation 2/LG8.heatMap.png]

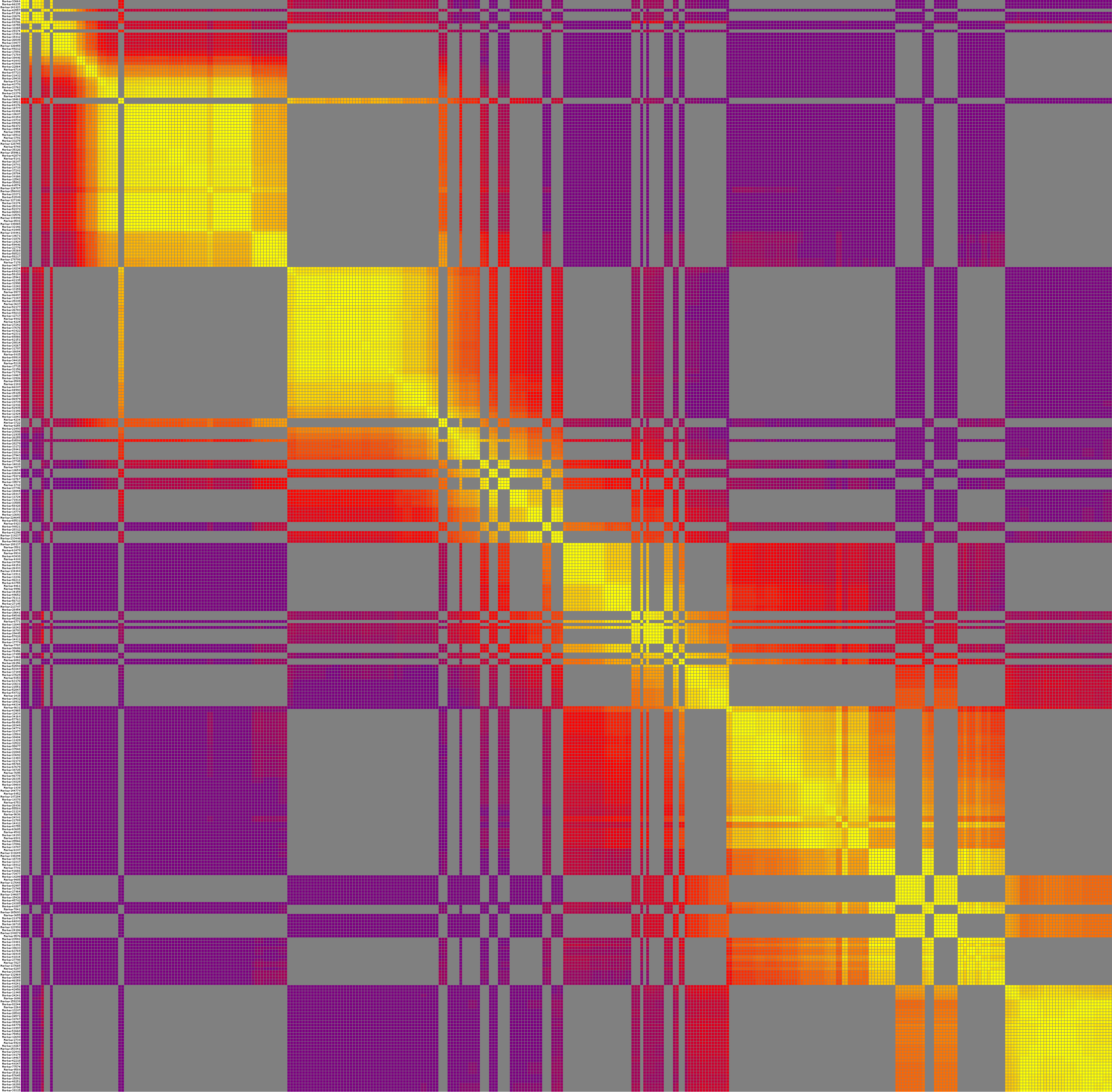

Supplement: Supplementary Material Presentation 2 — Heat map of the integrated maps. Markers of each row and column are ranked according to the map order; each small square represents the rate of recombination (r) between the two markers. [file Presentation2.ZIP › Supplementary Material Presentation 2/LG9.heatMap.png]
